# Spatiotemporal distribution and bivariate binary analysis of antenatal and delivery care utilizations in Ethiopia: EDHS 2000–2016

**DOI:** 10.1186/s12889-023-15369-5

**Published:** 2023-03-15

**Authors:** Shegaw Mamaru Awoke, Lijalem Melie Tesfaw, Muluwerk Ayele Derebe, Haile Mekonnen Fenta

**Affiliations:** 1 Departement of Statistics, Assosa University, Assosa, Ethiopia; 2 Departement of Statistics, Bahir Dar University, Bahir Dar, Ethiopia; 3 Epidemiology and Biostatistics Division, School of Public Health, University of Queensland, Brisbane, Queensland Australia

**Keywords:** Antenatal care, Delivery care, EDHS, Spatial, Ethiopia, Bivariate logistic

## Abstract

**Background:**

Antenatal care (ANC) is a maternal health care service given by skilled health professionals to pregnant women. Women may give birth at home or in health institutions. Home delivery care (DC) increases the likelihood of mortality of the mother and the newborn. Globally, each year nearly 303,000 maternal deaths occurred from complications of pregnancy and childbirth. Ethiopia alone accounted for 13,000 deaths, which disproportionately affects women living in different places of the country. Thus, this study aimed to assess the spatiotemporal patterns and associated factors of antenatal and delivery care utilization in Ethiopia.

**Method:**

This study used the 2000 to 2016 EDHS (Ethiopian and Demographic Health Survey) data as a source. A total weighted sample of 30,762 women (7966 in 2000, 7297 in 2005, 7908 in 2011, and 7591 in 2016) was used. The separate and bivariate logistic regression analyses with and without the spatial effect were modeled using SAS version 9.4 and ArcGIS version 10.8.

**Results:**

The spatial distribution of ANC and DC was non-random in Ethiopia. The overall odds ratio of ANC and DC was 2.09. In 2016, 31.8% and 33.2% of women had ANC and DC respectively. The estimated odds of following ANC among mothers from middle and rich households were 1.346 and 1.679 times the estimated odds of following ANC among mothers from poor households respectively. Women who had attained higher education were 1.56 and 2.03 times more likely to have ANC and DC respectively compared to women who had no formal education.

**Conclusions:**

Despite the government's report that women now have better access to maternal health care, a sizable proportion of women continue to give birth at home without going to the advised antenatal care appointment. Women and husbands with low education, having non-working partners, religion, regions of dwelling, residing in rural, lower birth order, low birth interval, unable to access mass media, low wealth status, and earlier EDHS survey years were significant predictors that hinder antenatal and delivery care utilization simultaneously in Ethiopia. Whereas the spatial variable significantly affects antenatal care and being unable to access mobile phones lead to low utilization of delivery care. We recommend that policymakers, planners, and researchers consider these variables and the spatiotemporal distribution of ANC and DC to reduce maternal mortality in Ethiopia. Besides, it is recommended that further studies use the latest EDHS survey data.

## Introduction

Women's delivery is the end of pregnancy and occurs when one or more infants leave the mother's body. Both home and hospital deliveries are choices available to women [1]. Home deliveries are non-skilled births that occurred outside of a medical facility, either with or without traditional birth attendants (TBAs). Institutional deliveries are births that occur in clinics or hospitals where women receive specialized care during childbirth from a medical expert [2]. A TBA is a woman who helps a delivery mother give birth at home. She first learned her trade by giving birth to her children or by working as an apprentice to other TBAs [2, 3].

To safeguard the health of both the mother and the unborn child, pregnant women need to get antenatal care (ANC), a maternal health service, from qualified medical professionals [4]. Prenatal care has the potential to reduce mother and child mortality, improve neonatal health, and minimize morbidity [4, 5]. The majority of maternal deaths worldwide, nearly 99%, occur in underdeveloped countries, mostly in sub-Saharan Africa and Southern Asia. These two regions account for roughly 86% of all maternal deaths worldwide, with sub-Saharan Africa alone accounting for 62% of those deaths [4]. According to a WHO report, only around 50% of deliveries in Africa were attended by qualified medical personnel, despite a worldwide increase from 58 to 68% from 2000 to 2008, respectively [6]. Maternal mortality is still a significant health issue in sub-Saharan Africa and other areas with little resources. A comprehensive growth and implementation of antenatal health care and institutional births could avoid or reduce such deaths [7].

Maternal mortality was 871, 673, and 420 per 100,000 live births in 2000, 2005, and 2016 EDHS, respectively. The fact that a sizable fraction of Ethiopian women lack access to health services or choose not to use them when they are available could be one explanation for the poor health condition of women in the nation [2]. By 2030, the Sustainable Development Goals aim to reduce maternal mortality from its current level of 216 deaths per 100,000 live births to 70 deaths per 100,000 live births [1]. Ethiopia continues to have a high rate of home deliveries, and only a small percentage of newborns are attended by trained medical personnel [2]. The proportion of births that takes place at health facilities and are attended by a skilled health professional has remained around 26 percent over the past five years a far lower level than in other African countries, Nigeria 41% and Cameroon 39% [8].

Prior research has attempted to pinpoint several variables influencing the use of prenatal care (ANC) and delivery care (DC) services. To our knowledge, the simultaneous geographical and temporal distribution of ANC and DC has not previously been studied [9]. The number of women who had at least one antenatal care visit and possible factors was summarized, but the spatial and temporal effect was not considered [10]. A spatial analysis was also performed by Yeneneh et al. [4] to investigate the utilization of ANC in Ethiopia without taking into account the temporal effect. On the other hand, a spatial and temporal analysis was used by [11] to determine the distribution and associated factors of home delivery in Ethiopia. However, they do not consider the distribution and determinants of antenatal care.

Several studies were conducted on antenatal and delivery care utilization and possible determinant factors in Ethiopia separately [10, 12]. However, maternal mortality can be reduced by securing ANC and DC jointly, which most of the previous studies failed to show. In addition, the space–time variation in ANC and DC utilization was also not addressed previously. The utilization of ANC and DC services varies in the country by changing its magnitude in space and time [13–16]. Therefore, this study intended to determine space–time patterns and possible factors that hinder the recommended ANC visit and institutional delivery among women in Ethiopia.

## Methods

### Study area

The study was conducted in Ethiopia. Ethiopia is the second most populous nation in Africa, with over 100 million people as of 2022 [2, 17]. Ethiopia has nine regional states and two administrative cities. Each Region is divided into zones and zones into administrative units called weredas [17].

### Data sources and study population

As the EDHS was first conducted in Ethiopia in 2000, in this study, the EDHS data for the four successive years 2000, 2005, 2011, and 2016 (without including the mini-2019 EDHS) was used. The survey includes pertinent social and health data, such as trends in population-wide critical health indicators and information on maternal and child health, that can be used to inform policy decisions. All Ethiopian women (15–49 years old) in the reproductive age range are considered as the study's population. Women in Ethiopia who had children within the previous five years of each survey for the most recent birth were involved in the study.

### Sample size and sampling procedure

A stratified two-stage cluster sampling procedure was used to select the nationally representative sample in all four surveys. In the first stage, a household listing operation was carried out in all the selected enumeration areas (EAs) for each survey year. In the second stage of choice, a total of 65,112 households (14,642 in 2000, 14,645 in 2005, 17,817 in 2011, and 18,008 in 2016) were selected with an equal probability of systematic selection from the newly created household list. Finally, a nationally representative sample of eligible 48,922 (15,367 in 2000, 14,070 in 2005, 16,515 in 2011, and 15,683 in 2016) women was interviewed [2].

**Fig. 1:**
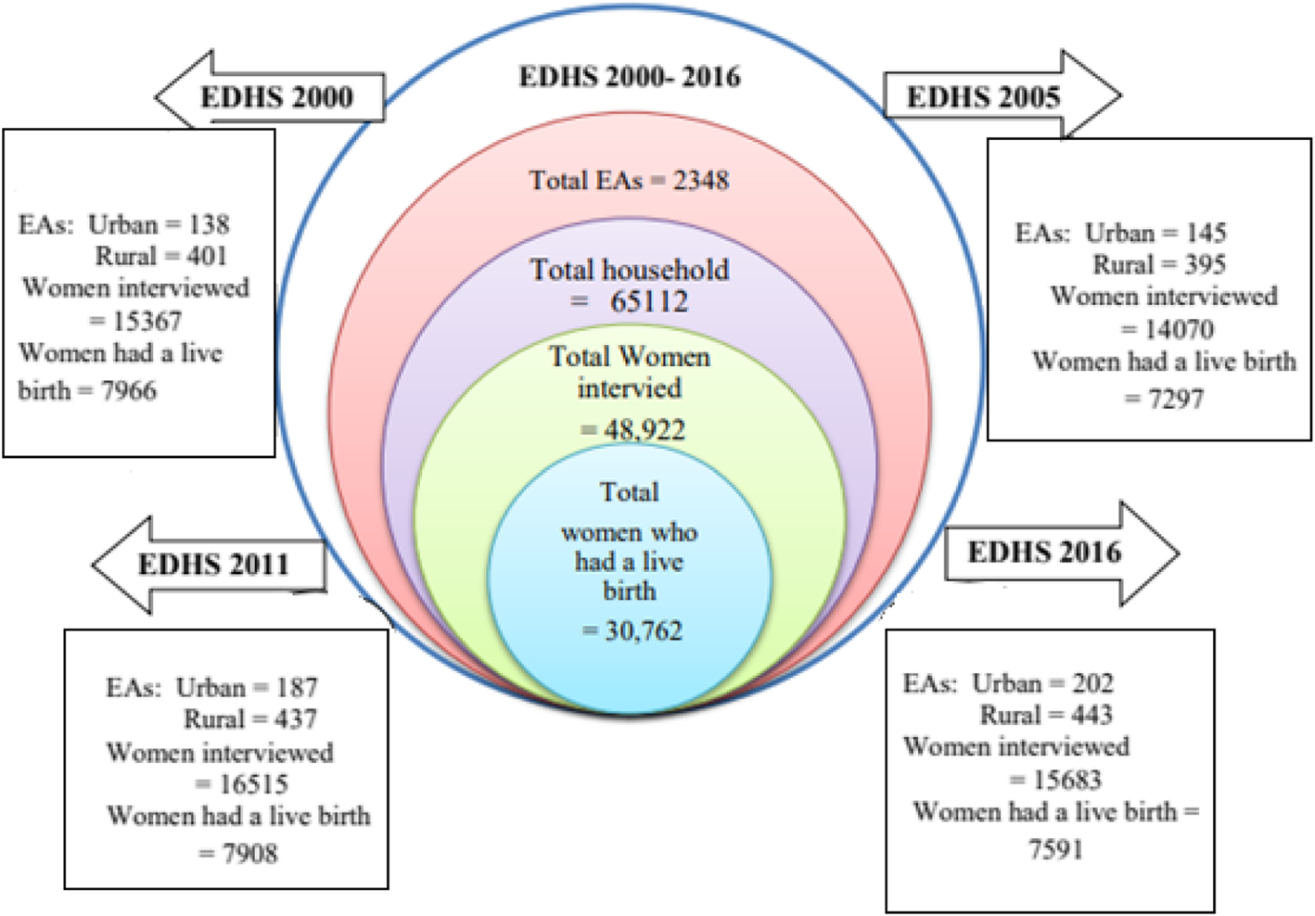
The flowchart for the data extraction procedure from EDHS 2000 to 2016

For this study, women who did not have a live birth in the five years before each survey were excluded. Therefore, the analytic sample for the current study consists of 30,762 women (7966 in 2000, 7297in 2005, 7908 in 2011, and 7591 in 2016) who had at least one live birth in the last five years before the survey (see Fig. 1). The data used for ANC and DC estimation were collected in the birth history section of the woman’s questionnaire, which was included in each of the four survey years.

### Study variables

#### Outcome variables

In this study, two binary outcome variables were considered. These are antenatal care (ANC) and delivery care (DC). Let Y_1_ and Y_2_denote the two response variables ANC and DC, respectively, then [18]:

$$\begin{array}{c}{\mathrm Y}_{1\mathrm i}=\left\{\begin{array}{c}Yes=1,if\,the\,\mathrm i^{\mathrm{th}}women\,have\,recommended\,four\,or\,more\,ANC\,visit\\No=0,otherwise\end{array}\right.\\And\\{\mathrm Y}_{2\mathrm i}=\left\{\begin{array}{c}Yes=1,if\,the\,\mathrm i^{\mathrm{th}}woman\,gave\,birth\,at\,a\,health\,facility\\No=0,if\,the\,\mathrm i^{\mathrm{th}}women\,gave\,birth\,a\,thome\end{array}\right.\end{array}$$

#### Independent variables

Based on the global and local literature reviews [2–8], all socio-demographic and obstetric characteristics that are associated with ANC and DC in the four-consecutive survey years were taken as independent variables (see Table 1).

**Table 1:** Variable description and coding

*Variables*	*Description and coding*
Age	Age of mother during the survey (1 = 15–19, 2 = 20–24, 3 = 25–29, 4 = 30–34, 5 = 35–39, 6 = 40–44, 7 = 45–49
Mother’s occupation	Employment status of mother (0 = not employed, 1 = employed)
Husband occupation	Employment status of husband (0 = not employed, 1 = employed)
mother’s education level	Current education level of the mother (0 = no education, 1 = primary, 2 = secondary, and 3 = higher)
Husband education level	Current education level of husband (0 = no education, 1 = primary, 2 = secondary and 3 = higher)
Wealth index	Household wealth index (1 = poor, 2 = medium, 3 = rich)
Marital status	Current marital status of mother (0 = unmarried, 1 = married)
Family size	Number of household members (1 = 1–3, 2 = 4–6, 3 = 7 and more)
Birth interval	Preceding birth interval in months (1 = < = 24, 2 = 25–36, 3 = > 36)
Birth order	Birth rank of child (1 = first, 2 = 2- 3 = 4–5, 4 = 6 and more)
Media exposure	Having access to mass media (0 = No, 1 = Yes)
Sex of household head	Sex of household head (1 = male, 2 = female)
Distance to the health Facility	Distance to health facility (1 = big problem, 2 = not a big problem)
Religion	Religion of mother (1 = Orthodox, 2 = Catholic, 3 = Protestant, 4 = Muslim, 5 = traditional/others)
Region	Mother’s place of Region(1 = Tigray, 2 = Afar, 3 = Amhara, 4 = Oromia, 5 = Somali, 6 = Benishangul, 7 = SNNP, 8 = Gambela, 9 = Harari, 10 = Addis Ababa, 11 = Dire Dawa)
Zone	Administrative zone of the mother
Time	The four Ethiopian Demographic and Health survey Years (EDHS 2000–2016)
*Si*	*The autocovariance variable*

#### Methods of statistical analysis

The data used for this study was obtained from four consecutive EDHS (2000 to 2016). Among a total of 30,762 women considered in this study, 7966, 7297, 7908, and 7591 of them were obtained from 2000, 2005, 2011, and 2016 EDHS respectively. Before fitting the model exploratory data analysis was performed. The Chi-square/OR test of association was carried out for the data to explore the relationship between the two outcome variables (ANC and DC) and each independent variable.

The data management was done using SPSS and STATA version 26. Whereas, the data were analyzed by the SAS version 9.4 with PROC LOGISTIC and PROC GLIMMIX by using the method of LAPLACE approximation while ArcGIS version 10.8 was used for mapping.

#### Spatial analysis and spatial autocorrelation

The statistical analysis of data that has a geographic label attached is the focus of the field of research known as spatial statistics. Nearby attribute values are more statistically dependent than far-away attribute values, which is the main characteristic of spatial statistical models [19]. Global indices of spatial autocorrelation are the summarization of the degree to which similar observations tend to occur near each other [20, 21].

#### Moran’s I

Moran's I is the basic extension of global indices of local autocorrelation. It is the similarity between areal units $i$ and $j$ is defined as the product of the respective difference between ${y}_{i}$ and ${y}_{j}$ with the overall mean divided by sample variance.

1 $$\mathrm{Moran}'sI=\frac{n\sum_i^n\sum_j^nwij(yi-\overline y)(yj-\overline y)}{\left(\sum_i^n\sum_j^nwij\right)\sum_i(yi-\overline y)^2}$$

where: Y_i_ represents the vector of observations at $\mathrm{n}$ different locations, and w_ij_ are elements of a spatial weight matrix.

2 $${W}_{ij}=\left\{\begin{array}{l}1,\text{ if two plot are adjacent }\\ 0,\text{ otherwise}\end{array}\right.$$

Assuming the weights W ji are binary, they simply identify which elements of the computation are to be included or excluded in the calculation [22].

The value of Moran's I vary in the interval$[-1 , 1]$. We can interpret the value as similar.to correlation coefficients. When the neighboring regions tend to have similar values, then the value of Moran's I will be positive and when the neighboring regions have dissimilar values then Moran's I will be negative [23].

#### Calculation of weight matrices (w_ij_)

Most of the spatial models are based on whether one region is the spatial neighbor of another region. The weight matrix is a square symmetric n x n matrix with (i,j) element equal to 1 if region i and j are neighbors of one another, and zero otherwise. The diagonal elements of the spatial weight matrix are zeros. Suppose an n × n spatial weighted matrix W, given by [24]:

3 $$Wij=\left[\begin{array}{ccccc}0& {w}_{12}& {w}_{13}& \cdots & {w}_{1N}\\ {w}_{21}& 0& {w}_{23}& \dots & {w}_{2N}\\ {w}_{31}& {w}_{32}& 0& \dots & {w}_{3N}\\ \vdots & \vdots & \vdots & \ddots & \vdots \\ {w}_{N1}& {w}_{N2}& {w}_{N3}& \cdots & 0\end{array}\right]$$

#### Hotspot and spatial interpolation

To be a statistically significant hot spot, a feature should have a high value and be surrounded by other features with high values as well [25, 26]. Spatial interpolation is a tool in GIS used to find the values of unknown points by estimating the values of properties at unsampled locations based on the set of observed values at known locations [27].

#### Kriging

Kriging is a geo-statistical method that uses known values and a semivariogram to predict the values at unmeasured locations [28]. The semi-variance is a measure of the degree of spatial dependence between samples. The magnitude of the semi-variance between points depends on the distance between the points. With kriging, therefore, predicted values are not the same as the “source” point but rather vary depending on their proximity to the source. The semivariogram model that best fits the data was developed to produce the optimum weights for interpolation [27, 28].

Kriging is most appropriate when we know there is a spatially correlated distance or directional bias in the data. It weights the surrounding measured values to derive a prediction for an unmeasured location. The general formula for the kriging interpolator is formed as a weighted sum of the data [27].

4 $$Z\left(s_0\right)={\textstyle\sum_{i=1}^n}\lambda_iZ\left(s_i\right)$$

where:

- Z(s_i_) = the measured value at the ith location.
- λ_i_ = an unknown weight for the measured value at i ^th^ location.
- s_0_ = the prediction location.
- n = the number of measured values.

#### Spatiotemporal analysis

All things are dynamic events, being, changing, and interacting with each other in space and time. Only by considering time and space together can we address how spatially coherent entities change over time or, in some cases, why they change. It turns out that a big part of the how and why of such change is due to interactions across space and time across multiple processes [29].

Spatiotemporal data is a simple extension of spatial data by adding a time dimension.

Spatiotemporal data are defined as [30]:

5 $$\mathbf{Y}(s,t)\equiv \left\{\mathbf{y}(s,t),(s,t)\in \mathbf{D}\in {\mathbf{R}}^{2}\times \mathbf{R}\right\}$$

where data is observed in n spatial areas or locations and at T time points. 

#### Binary logistic regression analysis

In binary logistic regression, the outcome variable Y_i_ (i = 1, 2,…,n) follows a Bernoulli probability distribution that takes on the value 1 with probability π_i_ and 0 with probability 1- π_i_. The relationship between ${\pi }_{i}$ and a vector of predictors for the i^th^ individual is given by [31, 32].

6 $$\begin{array}{c}\begin{array}{cc}\mathrm{logit}\left( {\uppi }_{i}\right)& =\mathrm{log}\left(\frac{{\pi }_{i}}{1-{\pi }_{i}}\right)\\ & \end{array} ={\beta }_{0}+{\beta }_{1}{X}_{i1}+{\beta }_{2}{X}_{i2}+\cdots +{\beta }_{k}{X}_{ik},i=\mathrm{1,2},\dots ,n\\ {\uppi }_{ i}=\frac{\mathrm{exp}({\beta }_{0}+{\beta }_{1}{X}_{i1}+{\beta }_{2}{X}_{i2}+\cdots +{\beta }_{k}{X}_{ik})}{1+ \mathrm{exp}({\beta }_{0}+{\beta }_{1}{X}_{i1}+{\beta }_{2}{X}_{i2}+\cdots +{\beta }_{k}{X}_{ik})}= \frac{\mathrm{exp}({X}^{,}{\beta }_{i})}{1+ \mathrm{exp}({X}^{,}{\beta }_{i})}\end{array}$$

where ${\beta }^{^{\prime}}s$ are the regression coefficient for the explanatory variables.

The odds are defined as the ratio between the probability of the occurrence of an event and the non-occurrence of an event, whereas the odds ratio is the ratio of two odds [33].

7 $$Odds= \frac{Pi}{1-Pi} \mathrm\,{and}\,Odds\,ratio=\frac{Odds\,1}{Odds\,2}$$

Adding the autocovariance transforms the linear predictor of the usual logistic regression model to consider the spatial effect:

8 $$\begin{array}{cc}\mathrm{logit}\left( {\uppi }_{i}\right)& =\mathrm{ log}\left(\frac{{\pi }_{i}}{1-{\pi }_{i}}\right)\\ & \end{array} ={\beta }_{0}+\beta X + \rho Si$$

where ${\beta }_{0}$ the coefficient for intercept, $\beta$ is a vector of coefficients for explanatory variables X; and ρ is the coefficient of the autocovariance variable.

The autocovariance variable (Si) at any site i is calculated as [34, 35]:

9 $${S}_{i}=\frac{{\sum }_{j=1}^{{k}_{i}} {w}_{ij}{y}_{j}}{{\sum }_{j=1}^{{k}_{i}} {w}_{ij}}$$

It is a weighted average of the geographic units among a set of Ki neighbors of the geographic unit i. Where ${y}_{j}$ is the response value of $y$ at site $j$ among the site i's set of Ki neighbors;${w}_{ij}$ is the element of the spatial weight matrix which is equal to 1 if regions i and j are neighbors and equal to zero otherwise.

#### Bivariate binary logistic regression

Bivariate logistic regression is an extension of univariate logistic regression when there are two categorical data of response variables and they are correlated to each other. In this study, each of the response variables has two categories. Let ${Y}_{1}$ and ${Y}_{2}$ be two response variables, then, the model can be shown in Table 2 and the joint probability of the response variables in Table 2 can be presented in Table 3 [36, 37].

**Table 2:** The (2 × 2) contingency table of the response variable

${Y}_{1}$	${Y}_{2}$		Total
	${Y}_{2}=1$	${Y}_{2}=0$	
${Y}_{1}=1$	${Y}_{11}$	${Y}_{10}$	${Y}_{1+}$
${Y}_{1}=0$	${Y}_{01}$	${Y}_{00}$	${Y}_{0+}=n-{Y}_{1+}$
Total	${Y}_{+1}$	${Y}_{+0}=n-{Y}_{+1}$	${Y}_{++}=n$

**Table 3:** The joint probability of the response variables

${Y}_{1}$	${Y}_{2}$		Total
	${Y}_{2}=1$	${Y}_{2}=0$	
${Y}_{1}=1$	${p}_{11}$	${p}_{10}$	${p}_{1+}$
${Y}_{1}=0$	${p}_{01}$	${p}_{00}$	${p}_{0+}=1-{p}_{1+}$
Total	${p}_{+1}$	${p}_{+0}=1-{p}_{+1}$	${p}_{++}=1$

Based on Tables 2 and 3, the random variables ${Y}_{11},{Y}_{10},{Y}_{01}$, and ${Y}_{00}$ follows the multinomial distribution with a joint probability function defined by:

10 $${\varvec{P}}\left({{\varvec{Y}}}_{11}={{\varvec{y}}}_{11},{{\varvec{Y}}}_{10}={{\varvec{y}}}_{10},{{\varvec{Y}}}_{01}={{\varvec{y}}}_{01},\boldsymbol{ }\boldsymbol{ }\boldsymbol{ }{{\varvec{Y}}}_{00}=\boldsymbol{ }{{\varvec{y}}}_{00}\right)=\prod_{{\varvec{g}}=0}^{1} \prod_{{\varvec{h}}=0}^{1} \frac{{{\varvec{p}}}_{{\varvec{g}}{\varvec{h}}}^{{{\varvec{y}}}_{{\varvec{g}}{\varvec{h}}}}}{{{\varvec{y}}}_{{\varvec{g}}{\varvec{h}}}!},0<{{\varvec{p}}}_{{\varvec{g}}{\varvec{h}}\boldsymbol{ }\boldsymbol{ }}<1$$

where: $$g,h=\mathrm{0,1};{y}_{gh}=\mathrm{0,1};{y}_{00}=1-{y}_{11}-{y}_{10}-{y}_{01}$$ and $${p}_{00}=1-{p}_{11}-{p}_{10}-{p}_{01}$$.

## Result

### EDHS 2000

The association between the predictor variable and the two response variables (ANC and DC) in EDHS 2000 was revealed in Table 4. The chi-square statistics presented in the table indicate that both ANC and DC were significantly associated with the predictor variables such as mother’s education, husband's education, religion, region, place of residence, birth order, wealth index, and access to mass media, (*P*-value <  = 0.05).

**Table 4:** Association of socio-demographic and obstetric characteristics with ANC and DC, EDHS 2000

*Variables*	*Categories*	*Weighted Frequency (%)*	*Antenatal care visit*			*Place of Delivery*		
			Yes (%)	No (%)	X^2^-*p* value	Health facility (%)	Home (%)	X^2^-*p* value
Age	15–19	473(5.9)	45 (5.4)	428(6.0)	0.000	45(10.2)	428(5.7)	0.000
	20–24	1727(21.7)	179(21.5)	1548(21.7)		93(21.0)	1634(21.7)	
	25–29	2021(25.4)	264(31.7)	1757(24.6)		146(33.0)	1882(25.0)	
	30–34	1493(18.7)	156(18.8)	1337(18.7)		70(15.8)	1426(18.9)	
	35–39	1219(15.3)	119(14.3)	1100(15.5)		51(11.5)	1168(15.5)	
	40–44	706(8.9)	48(5.8)	658(9.2)		26(5.9)	680(9.0)	
	45–49	329(4.1)	21(2.5)	308(4.3)		12(2.7)	318(4.2)	
Mother’s education	No education	6539(82.1)	403(48.5)	6136(86.0)	0.000	153(34.6)	6397(84.9)	0.000
	Primary	1003(12.6)	219(26.4)	784(11.0)		99(22.4)	904(12.0)	
	Secondary	400(5.0)	192(23.1)	208(2.9)		172(38.9)	228(3.0)	
	Higher	24(0.3)	17(2.0)	7(0.1)		18(4.1)	7(0.1)	
Mother’s Occupation	Not working	2781(34.9)	306(36.8)	2475(34.7)	0.233	165(37.3)	2619(34.8)	0.271
	Working	5184(65.1)	526(63.2)	4658(65.3)		227(62.7)	4915(65.2)	
Husband education	No education	5155(65.3)	288(35.0)	4867(68.8)	0.000	85(20.1)	5071(67.8)	0.000
	Primary	1894(24.0)	222(27.0)	1672(23.6)		117(27.7)	1777(23.8)	
	Secondary	736(9.3)	246(29.9)	490(6.9)		169(39.9)	566(7.6)	
	Higher	115(1.5)	66(8.0)	49(0.7)		52(12.3)	63(0.8)	
Husband occupation	Not working	38(0.5)	12(1.5)	27(0.4)	0.000	3(0.7)	35(0.5)	0.000
	Working	7857(99.5)	810(98.5)	7048(99.6)		419(99.3)	7438(99.5)	
Household head	Male	6754(84.8)	680(81.8)	6074(85.1)	0.012	329(74.8)	6425(85.4)	0.000
	Female	1212(15.2)	151(18.2)	1061(14.9)		111(25.2)	1101(14.6)	
Marital status	Unmarried	783(9.8)	97(11.7)	686(9.6)	0.059	90(20.5)	693(9.2)	0.000
	Married	7184(90.2)	734(88.3)	6450(90.4)		350(79.5)	6834(90.8)	
Religion	Orthodox	4053(50.9)	458(55.0)	3596(50.4)	0.000	285(64.8)	3768(50.1)	0.000
	Catholic	59(0.7)	6(0.7)	53(0.7)		4(0.9)	55(0.7)	
	Protestant	1228(15.4)	87(10.5)	1142(16.0)		52(11.8)	1176(15.6)	
	Muslim	2334(29.3)	272(32.7)	2062(28.9)		95(21.6)	2239(29.7)	
	Others	293(3.7)	9(1.1)	284(4.0)		4(0.9)	289(3.8)	
Region	Tigray	537(6.7)	81(9.7)	456(6.4)	0.000	27(6.1)	510(6.8)	0.000
	Afar	84(1.1)	6(0.7)	78(1.1)		5(1.1)	79(1.0)	
	Amhara	2222(27.9)	104(12.5)	2118(29.7)		81(18.4)	2141(28.4)	
	Oromo	3057(38.4)	324(38.9)	2733(38.3)		122(27.7)	2935(39.0)	
	Somalia	84(1.1)	3(0.4)	82(1.1)		5(1.1)	79(1.0)	
	Benishangul	82(1.0)	9(1.1)	73(1.0)		5(1.1)	73(1.0)	
	SNNP	1689(21.2)	179(21.5)	1510(21.2)		9(2.0)	1617(21.5)	
	Gambela	22(0.3)	7(0.8))	15(0.2)		72(16.4)	17(0.2)	
	Harari	16(0.2)	4(0.5)	12(0.2)		5(1.1)	11(0.1)	
	Addis Ababa	148(1.9)	106(12.7)	41(0.6)		100(22.7)	48(0.6)	
	Dire Dwa	27(0.3)	9(1.1)	18(0.3)		9(2.0)	18(0.2)	
Residence	Urban	905(11.4)	396(47.7)	509(7.1)	0.000	302(68.8)	603(8.0)	0.000
	Rural	7061(88.6)	435(52.3)	6626(92.9)		137(31.2)	6924(92.0)	
Family size	1–3	1112(14.0)	118(14.2)	994(13.9)	0.318	89(20.3)	1023(13.6)	0.000
	4–6	4039(50.7)	402(48.3)	3637(51.0)		205(46.7)	3834(50.9)	
	7 and above	2816(35.3)	312(37.5)	2504(35.1)		145(33.0)	2671(35.5)	
Birth order	First	1362(17.1)	197(23.7)	1165(16.3)	0.000	171(39.0)	1191(15.8)	0.000
	2–3	2366(29.7)	265(31.9)	2101(29.4)		131(29.8)	2235(29.7)	
	4–5	1702(21.4)	207(24.9)	1495(21.0)		72(16.4)	1630(21.7)	
	6 and above	2536(31.8)	161(19.4)	2375(33.3)		65(14.8)	2471(32.8)	
Birth interval	< = 24	1290(19.6)	114(18.2)	1176(19.7)	0.140	47(17.8)	1243(19.6)	0.214
	25–36	2451(37.2)	219(34.9)	2232(37.4)		89(33.7)	2362(37.3)	
	> = 37	2850(43.2)	295(47.0)	2556(42.9)		128(48.5)	2722(43.0)	
Mass media	No	5795(72.8)	336(40.5)	5459(76.6)	0.000	114(26.0)	5681(75.5)	0.000
	Yes	2163(27.2)	494(59.5)	1669(23.4)		324(74.0)	1839(24.5)	
Has mobile/Telephone	No	7921(99.3)	791(95.2)	7118(99.8)	0.000	404(91.6)	7517(99.7)	0.000
	Yes	56(0.7)	40(4.8)	16(0.2)		37(8.4)	19(0.3)	
Wealth index	Poor	3577 (44.9)	182(21.9)	3394(47.6)	0.000	134(30.5)	3442(45.7)	0.000
	Middle	999 (12.5)	62(7.5)	937(13.1)		36(8.2)	963(12.8)	
	*Rich*	*3391 (42.6)*	*587(70.6)*	*2804(39.3)*		*269(61.3)*	*3122(41.5)*	

The majority of the respondents in 2021(25.4%) were aged 25–29 years and the maximum prevalence of both ANC and DC were observed in this age group (31.7 and 33.0 percent respectively). Among the total number of respondents, 82.1%, 12.6%, 5.0, and 0.3% were non-educated, primary educated, secondary educated, and higher educated respectively, and among those who had attended antenatal care, 48.5, 26.4, 23.1 and 2.0% had no, primary, secondary and higher education respectively. Regarding religion, the prevalence of both ANC and DC was maximum among orthodox followers and minimum among Catholic followers (see Table 4).

### EDHS 2005

In the second EDHS (EDHS 2005), all predictors under study were significantly associated with antenatal care visits and only the husband's occupation was insignificantly associated with the place of delivery. The minimum and maximum prevalence of both ANC and DC were reported among women of age 25–29 and 45–49 years respectively. The prevalence of home delivery decreases as the education level of mothers and their partners increases. This prevalence was 81.7, 15.8, 2.4, and 0.1 percent among women who attend no formal education, primary education, secondary and higher education respectively (see Table 5).

**Table 5:** Association of socio-demographic and obstetric characteristics with ANC and DC, EDHS 2005

*Variables*	*Categories*	*Weighted frequency (%)*	*Antenatal care Visit*			*Place of Delivery*		
			Yes (%)	No (%)	X^2^-*p* value	Health facility (%)	Home (%)	X^2^-*p* value
Age	15–19	439(6.0)	49(5.5)	390(6.1)	0.000	26(5.2)	413(6.1)	0.000
	20–24	1473(20.2)	233(26.2)	1240(19.3)		137(27.6)	1336(19.6)	
	25–29	1960(26.9)	256(28.8)	1704(26.6)		164(33.1)	1795(26.4)	
	30–34	1425(19.5)	182(20.5)	1243(19.4)		78(15.7)	1347(19.8)	
	35–39	1135(15.6)	92(10.3)	1043(16.3)		46(9.3)	1089(16.0)	
	40–44	576(7.9)	56(6.3)	520(8.1)		34(6.9)	542(8.0)	
	45–49	290(4.0)	21(2.4)	269(4.2)		11(2.2)	278(4.1)	
Mother’s education	No education	5726(78.5)	453(51.0)	5273(82.3)	0.000	170(34.3)	5556(81.7)	0.000
	Primary	1202(16.5)	203(22.8)	999(15.6)		126(25.4)	1077(15.8)	
	Secondary	328(4.5)	201(22.6)	127(2.0)		168(33.9)	160(2.4)	
	Higher	40(0.5)	32(3.6)	8(0.1)		32(6.5)	7(0.1)	
Mother’s Occupation	Not working	5033(69.1)	522(59.0)	4511(70.5)	0.000	294(59.3)	4739(69.8)	0.000
	Working	2254(30.9)	363(41.0)	1891(29.5)		202(40.7)	2052(30.2)	
Husband education	No education	4295(59.3)	284(32.3)	4011(63.1)	0.000	104(21.2)	4190(62.1)	0.000
	Primary	2107(29.1)	258(29.4)	1849(29.1)		121(24.7)	1986(29.4)	
	Secondary	736(10.2)	274(31.2)	462(7.3)		203(41.4)	534(7.9)	
	Higher	99(1.4)	62(7.1)	37(0.6)		62(12.7)	38(0.6)	
Husband occupation	Not working	56(0.8)	20(2.3)	36(0.6)	0.000	5(1.0)	51(0.8)	0.508
	Working	7187(99.2)	856(97.7)	6331(99.4)		482(99.0)	6705(99.2)	
Household head	Male	6409(87.8)	710(79.9)	5699(88.9)	0.000	383(77.2)	6026(88.6)	0.000
	Female	887(12.2)	179(20.1)	708(11.1)		113(22.8)	774(11.4)	
Marital status	Unmarried	535(7.3)	88(9.9)	447(7.0)	0.002	61(12.3)	474(7.0)	0.000
	Married	6761(92.7)	800(90.1)	5961(93.0)		435(87.7)	6326(93.0)	
Religion	Orthodox	3259(44.7)	471(53.0)	2788(43.5)	0.000	297(60.0)	2962(43.6)	0.000
	Catholic	75(1.0)	3(0.3)	72(1.1)		2(0.4)	73(1.1)	
	Protestant	1403()19.2	158(17.8)	1245(19.4)		93(18.8)	1311(19.3)	
	Muslim	2374(32.5)	247(27.8)	2127(33.2)		92(18.6)	2282(33.6)	
	Others	184(2.5)	9(1.0)	175(2.7)		11(2.2)	173(2.5)	
Region	Tigray	480(6.6)	86(9.7)	394(6.1)	0.000	37(7.4)	443(6.5)	0.000
	Afar	68(0.9)	5(0.6)	63(1.0)		3(0.6)	65(1.0)	
	Amhara	1856(25.4)	135(15.2)	1721(26.9)		78(15.7)	1777(26.1)	
	Oromo	2714(37.2)	274(30.9)	2440(38.1)		147(29.6)	2568(37.8)	
	Somalia	288(3.9)	13(1.5)	275(4.3)		19(3.8)	269(4.0)	
	Benishangul	68(0.9)	7(0.8)	61(1.0)		14(2.8)	54(0.8)	
	SNNP	1630(22.3)	245(27.6)	1385(21.6)		76(15.3)	1555(22.9)	
	Gambela	23(0.3)	6(0.7)	17(0.3)		5(1.0)	18(0.3)	
	Harari	15(0.2)	4(0.5)	11(0.2)		6(1.2)	9(0.1)	
	Addis Ababa	129(1.8)	104(11.7)	25(0.4)		104(20.9)	25(0.4)	
	Dire Dawa	24(0.3)	8(0.9)	16(0.2)		8(1.6)	17(0.3)	
Residence	Urban	633(8.7)	346(38.9)	287(4.5)	0.000	292(59.0)	341(5.0)	0.000
	Rural	6663(91.3)	543(61.1)	6120(95.5)		203(41.0)	6460(95.0)	
Family size	1–3	907(12.4)	149(16.8)	758(11.8)	0.000	123(24.8)	784(11.5)	0.000
	4–6	3637(49.8)	470(52.9)	3167(49.4)		246(49.6)	3390(49.9)	
	7 and above	2752(37.7)	269(30.3)	2483(38.7)		127(25.6)	2626(38.6)	
Birth order	First	1190(16.3)	226(25.4)	964(15.0)	0.000	197(39.8)	992(14.6)	0.000
	2–3	2087(28.6)	340(38.2)	1747(27.3)		171(34.5)	1915(28.2)	
	4–5	1692(23.2)	148(16.6)	1544(24.1)		58(11.7)	1634(24.0)	
	6 and above	2328(31.9)	175(19.7)	2153(33.6)		69(13.9)	2259(33.2)	
Birth interval	< = 24	1264(20.7)	122(18.4)	1142(21.0)	0.001	55(18.5)	1209(20.8)	0.000
	25–36	2001(32.8)	186(28.1)	1815(33.4)		70(23.6)	1930(33.2)	
	> = 37	2838(46.5)	354(53.5)	2484(45.7)		172(57.9)	2666(45.9)	
Media exposure	No	4556(62.7)	305(34.5)	4251(66.6)	0.000	122(24.7)	4434(65.5)	0.000
	Yes	2710(37.3)	580(65.5)	2130(33.4)		372(75.3)	2338(34.5)	
Has mobile/Telephone	No	7147(98.0)	780(87.8)	6367(99.4)	0.000	396(80.0)	6751(99.3)	0.000
	Yes	147(2.0)	108(12.2)	39(0.6)		99(20.0)	47(0.7)	
Wealth index	Poor	3066(42.0)	156(17.6)	2910(45.4)	0.000	47(9.5)	3019(44.4)	0.000
	Middle	1585(21.7)	135(15.2)	1450(22.6)		31(6.3)	1554(22.9)	
	Rich	2644(36.2)	596(67.2)	2048(32.0)		417(84.2)	2227(32.8)	
Distance to health facility	Not big problem	4588(62.9)	671(75.6)	3917(61.1)	0.000	363(73.2)	4225(62.1)	0.000
	*Big problem*	*2707(37.1)*	*217(24.4)*	*2490(38.9)*		*133(26.8)*	*2575(37.9)*	

### EDHS 2011

Table 6 shows the association of ANC and DC with the socio-demographic and obstetric characteristics of women in the 2011 EDHS. Judging by the chi-square p-value, most predictors except marital status were significantly associated with antenatal care visits and all predictors except husband occupation were significantly associated with place of delivery. The prevalence of ANC and DC were examined across the different characteristics of women and both ANC and DC were more practiced among women aged 25–29 years compared to other age groups. A high proportion of home delivery and fewer ANC visit was reported among women not working compared to those working (see Table 6).

**Table 6:** Association of socio-demographic and obstetric characteristics with ANC and DC, EDHS 2011

*Variables*	*Categories*	*Weighted frequency (%)*	*Antenatal care visit*			*Place of Delivery*		
			Yes (%)	No (%)	X^2^-*p* value	Health facility (%)	Home (%)	X^2^-*p* value
Age	15–19	402(5.1)	59(3.8)	343(5.4)	0.000	42(4.4)	360(5.2)	0.000
	20–24	1608(20.3)	335(21.8)	1273(20.0)		275(28.6)	1333(19.2)	
	25–29	2383(30.1)	516(33.6)	1867(29.3)		344(35.8)	2038(29.3)	
	30–34	1489(18.8)	262(17.1)	1227(19.3)		152(15.8)	1337(19.2)	
	35–39	1239(15.7)	257(16.7)	982(15.4)		95(9.9)	1144(16.5)	
	40–44	572(7.2)	84(5.5)	488(7.7)		34(3.5)	537(7.7)	
	45–49	216(2.7)	22(1.4)	194(3.0)		18(1.9)	198(2.9)	
Mother’s education	No education	5270(66.6)	665(43.3)	4605(72.3)	0.000	275(28.6)	4995(71.9)	0.000
	Primary	2270(28.7)	630(41.0)	1640(25.7)		415(43.2)	1855(26.7)	
	Secondary	226(2.9)	147(9.6)	79(1.2)		160(16.7)	65(0.9)	
	Higher	142(1.8)	93(6.1)	49(0.8)		110(11.5)	32(0.5)	
Mother’s Occupation	Not working	3509(44.8)	607(39.9)	2902(46.0)	0.000	418(44.0)	3092(44.9)	0.619
	Working	4325(55.2)	914(60.1)	3411(54.0)		531(56.0)	3794(55.1)	
Husband education	No education	3918(50.0)	464(30.5)	3454(54.7)	0.000	185(19.7)	3734(54.2)	0.000
	Primary	3183(40.7)	706(46.4)	2477(39.3)		397(42.3)	2785(40.4)	
	Secondary	433(5.5)	173(11.4)	260(4.1)		180(19.2)	253(3.7)	
	Higher	296(3.8)	177(11.6)	119(1.9)		177(18.8)	119(1.7)	
Husband occupation	Not working	64(0.8)	25(1.6)	39(0.6)	0.000	10(1.1)	54(0.8)	0.371
	Working	7732(99.2)	1494(98.4)	6238(99.4)		926(98.9)	6807(99.2)	
Household head	Male	6611(83.6)	1237(80.6)	5374(84.3)	0.000	696(72.5)	5915(85.1)	0.000
	Female	1297(16.4)	298(19.4)	999(15.7)		264(27.5)	1033(14.9)	
Marital status	Unmarried	723(9.1)	138(9.0)	585(9.2)	0.817	122(12.7)	600(8.6)	0.000
	Married	7185(90.9)	1397(91.0)	5788(9.8)		838(87.3)	6347(91.4)	
Religion	Orthodox	3327(42.1)	775(50.5)	2552(40.1)	0.000	580(60.4)	2747(39.6)	0.000
	Catholic	81(1.0)	15(1.0)	66(1.0)		17(1.8)	64(0.9)	
	Protestant	1763(22.3)	292(19.0)	1471(23.1)		164(17.1)	1599(23.0)	
	Muslim	2563(32.4)	436(28.4)	2127(33.4)		196(20.4)	2367(34.1)	
	Others	169(2.1)	17(1.1)	152(2.4)		3(0.3)	166(2.4)	
Region	Tigray	530(6.7)	164(10.7)	366(5.7)	0.000	76(7.9)	454(6.5)	0.000
	Afar	78(1.0)	9(0.6)	69(1.1)		6(0.6)	72(1.0)	
	Amhara	1991(25.2)	266(17.3)	1725(27.1)		220(22.9)	1772(25.5)	
	Oromo	3116(39.4)	579(37.7)	2537(39.8)		303(31.5)	2814(40.5)	
	Somalia	198(2.5)	14(0.9)	184(2.9)		18(1.9)	179(2.6)	
	Benishangul	92(1.2)	15(1.0)	77(1.2)		17(1.8)	75(1.1)	
	SNNP	1634(20.7)	293(19.1)	1341(21.0)		133(13.8)	1501(21.6)	
	Gambela	31(0.4)	10(0.7)	21(0.3)		9(0.9)	21(0.3)	
	Harari	19(0.2)	7(0.5)	12(0.2)		8(0.8)	12(0.2)	
	Addis Ababa	192(2.4)	168(10.9)	24(0.4)		158(16.4)	34(0.5)	
	Dire Dwa	27(0.3)	11(0.7)	16(0.3)		13(1.4)	14(0.2)	
Residence	Urban	1188(15.0)	562(36.6)	626(9.8)	0.000	630(65.6)	558(8.0)	0.000
	Rural	720(85.0)	973(63.4)	5747(90.2)		331(34.4)	6389(92.0)	
Family size	1–3	1079(13.7)	294(19.2)	785(12.3)	0.000	255(26.5)	825(11.9)	0.000
	4–6	4057(51.3)	801(52.2)	3256(51.1)		514(53.5)	3543(51.0)	
	7 and above	2771(35.0)	440(28.7)	2331(36.6)		192(20.0)	2580(37.1)	
Birth order	First	1399(17.7)	390(25.4)	1009(15.8)	0.000	371(38.6)	1028(14.8)	0.000
	2–3	2462(31.1)	503(32.8)	1959(30.7)		365(38.0)	2097(30.2)	
	4–5	1814(22.9)	346(22.5)	1468(23.0)		128(13.3)	1686(24.3)	
	6 and above	2233(28.2)	296(19.3)	1937(30.5)		97(10.1)	21.36(30.7)	
Birth interval	< = 24	1178(18.1)	155(13.6)	1023(19.1)	0.000	94(16.1)	1084(18.3)	0.000
	25–36	2262(34.8)	315(27.7)	1947(36.3)		103(17.6)	2160(36.5)	
	> = 37	3057(47.1)	667(58.7)	2390(44.6)		388(66.3)	2669(45.1)	
Mass media	No	3171(40.2)	277(18.0)	2894(45.5)	0.000	138(14.4)	3033(43.8)	0.000
Has mobile/Telephone	Yes	4720(59.8)	1258(82.0)	3462(54.5)		822(85.6)	3898(56.2)	
	No	7729(97.8)	1410(92.0)	6319(99.2)	0.000	827(86.1)	6902(99.4)	0.000
	Yes	177(2.2)	123(8.0)	54(0.8)		134(13.9)	44(0.6)	
Wealth index	Poor	3435(43.4)	347(22.6)	3088(48.5)	0.000	112(11.7)	3324(47.8)	0.000
	Middle	1628(20.6)	226(14.7)	1402(22.0)		65(6.8)	1563(22.5)	
	Rich	2844(36.0)	962(62.7)	1882(29.5)		783(81.6)	2061(29.7)	
Distance to health facility	Not big problem	5403(68.4)	1218(79.5)	4185(65.7)	0.000	796(83.4)	4606(66.3)	0.000
	Big problem	2499(31.6)	314(20.5)	2185(34.3)		158(16.6)	2340(33.7)	
Insurance	Not insured	7848(99.3)	1500(97.8)	6348(99.7)	0.000	925(96.7)	6923(99.7)	0.000
	*insured*	*54(0.7)*	*34(2.2)*	*20(0.7)*		*32(3.3)*	*22(0.3)*	

### EDHS 2016

Likewise in the 2016 EDHS, presented in Table 7, all predictors in the study except sex of household head had a statistically significant association with antenatal care, and all predictors except marital status were significantly associated with antenatal care visits and all predictors under study were significantly associated with place of delivery. Regarding mothers' age, the highest proportion of ANC and DC were reported among mothers in the age groups 25–29. Among women who do not have the recommended antenatal care visit, 70.3% were women with no formal education and only 1.2% were women who attend higher education. Among women who attend the recommended antenatal care visit, 29.9%, 18.5%, and 51.6% have poor, middle, and rich wealth indexes respectively (see Table 7).

**Table 7:** Association of socio-demographic and obstetric characteristics with ANC and DC, EDHS 2016

*Variables*	*Categories*	*Weighted frequency (%)*	*Antenatal care visit*			*Place of Delivery*		
			Yes (%)	No (%)	X^2^-*p* value	Health facility (%)	Home (%)	X^2^-*p *value
Age	15–19	339(4.5)	104(4.3)	235(4.5)	0.000	145(5.7)	194(3.8)	0.000
	20–24	1465(19.3)	454(18.8)	1011(19.5)		600(23.8)	865(17.1)	
	25–29	2165(28.5)	791(32.8)	1374(26.6)		778(30.8)	1387(27.4)	
	30–34	1661(21.9)	536(22.2)	1125(21.7)		499(19.8)	1162(22.9)	
	35–39	1206(15.9)	343(14.2)	863(16.7)		360(14.3)	846(16.7)	
	40–44	546(7.2)	144(6.0)	402(7.8)		109(4.3)	437(8.6)	
	45–49	207(2.7)	42(1.7)	164(3.2)		32(1.3)	175(3.5)	
Mother’s education	No education	4791(63.1)	1156(47.9)	3635(70.3)		1036(41.1)	3755(74.1)	0.000
	Primary	2149(28.3)	828(34.3)	1321(25.5)		927(36.7)	1222(24.1)	
	Secondary	419(5.5)	263(10.9)	156(3.0)		346(13.7)	74(1.5)	
	Higher	229(3.0)	167(6.9)	62(1.2)		214(8.5)	16(0.3)	
Mother’s Occupation	Not working	4078(53.7)	1206(50.0)	2872(55.5)	0.000	1238(49.0)	2840(56.1)	0 .000
	Working	3511(46.3)	1208(50.0)	2303(44.5)		1286(51.0)	2226(43.9)	
Husband education	No education	3389(47.7)	788(35.2)	2601(53.5)	0.000	750(32.0)	2639(55.4)	0.000
	Primary	2731(38.4)	908(40.5)	1823(37.5)		871(37.2)	1860(39.0)	
	Secondary	612(8.6)	311(13.9)	301(6.2)		409(17.7)	204(4.3)	
	Higher	375(5.3)	234(10.4)	141(2.9)		313(13.4)	63(1.3)	
Husband occupation	Not working	571(8.0)	122(5.4)	449(9.2)	0.000	154(6.6)	417(8.7)	0.002
	Working	6538(92.0)	2120(94.6)	4418(90.8)		2189(93.4)	4349(91.3)	
Household head	Male	6473(85.3)	2018(83.6)	4455(86.1)	0.004	2083(82.6)	4390(86.7)	0.000
	Female	1116(14.7)	396(16.4)	720(13.9)		440(17.4)	676(13.3)	
Marital status	Unmarried	481(6.3)	173(7.2)	308(6.0)	0.044	181(7.2)	300(5.9)	0.035
	Married	7109(93.7)	2242(92.8)	4867(94.0)		2343(92.8)	4766(94.1)	
Religion	Orthodox	2882(38.0)	1124(46.5)	1758(34.0)	0.000	1226(48.6)	1656(32.7)	0.000
	Catholic	72(0.9)	20(0.8)	52(1.0)		13(0.5)	58(1.1)	
	Protestant	1652(21.8)	526(21.8)	1126(21.8)		501(19.9)	1150(22.7)	
	Muslim	2824(37.2)	726(30.1)	2098(40.5)		766(30.4)	2058(40.6)	
	Others	160(2.1)	19(0.8)	141(2.7)		17(0.7)	143(2.8)	
Region	Tigray	537(7.1)	304(12.6)	233(4.5)	0.000	357(14.1)	180(3.6)	0.000
	Afar	71(0.9)	15(0.6)	56(1.1)		14(0.6)	57(1.1)	
	Amhara	1633(21.5)	514(21.3)	1119(21.6)		516(20.5)	1116(22.0)	
	Oromo	3129(41.2)	692(28.6)	2437(47.1)		793(31.4)	2337(46.1)	
	Somalia	269(3.5)	32(1.3)	237(4.6)		53(2.1)	215(4.2)	
	Benishangul	81(1.1)	34(1.4)	47(0.9)		27(1.1)	54(1.1)	
	SNNP	1600(21.1)	611(25.3)	989(19.1)		530(21.0)	1070(21.1)	
	Gambela	21(0.3)	9(0.4)	12(0.2)		10(0.4)	11(0.2)	
	Harari	17(0.2)	6(0.2)	11(0.2)		10(0.4)	7(0.1)	
	Addis Ababa	199(2.6)	177(7.3)	22(0.4)		191(7.6)	7(0.1)	
	Dire Dwa	33(0.4)	22(0.9)	11(0.2)		22(0.9)	12(0.2)	
Residence	Urban	969(12.8)	608(25.2)	361(7.0)	0.000	816(32.3)	153(3.0)	0.000
	Rural	6621(87.2)	1807(74.8)	4814(93.0)		1707(67.7)	4914(97.0)	
Family size	1–3	1033(13.6)	397(16.4)	636(12.3)	0.000	517(20.5)	515(10.2)	0.000
	4–6	3889(51.2)	1313(54.4)	2576(49.8)		1297(51.4)	2591(51.1)	
	7 and above	2668(35.2)	705(29.2)	1963(37.9)		709(28.1)	1960(38.7)	
Birth order	First	1434(18.9)	602(24.9)	832(16.1)	0.000	815(32.3)	619(12.2)	0.000
	2–3	2282(30.1)	808(33.5)	1474(28.5)		855(33.9)	1426(28.1)	
	4–5	1751(23.1)	524(21.7)	1227(23.7)		440(17.4)	1312(25.9)	
	6 and above	2123(28.0)	481(19.9)	1642(31.7)		413(16.4)	1709(33.7)	
Birth interval	< = 24	1255(20.4)	306(16.9)	949(21.9)	0.000	253(14.8)	1002(22.6)	0.000
	25–36	1873(30.5)	467(25.8)	1406(32.4)		418(24.5)	1456(32.8)	
	> = 37	3016(49.1)	1034(57.2)	1982(45.7)		1033(60.6)	1983(44.7)	
Mass media	No	4969(65.5)	1241(51.4)	3728(72.0)	0.000	1194(47.3)	3775(74.5)	0.000
	Yes	2621(34.5)	1174(48.6)	1447(28.0)		1329(52.7)	1291(25.5)	
Has mobile/Telephone	No	6176(81.4)	1625(67.3)	4551(87.9)	0.000	1602(63.5)	4574(90.3)	0.000
	Yes	1413(18.6)	789(32.7)	624(12.1)		921(36.5)	492(9.7)	
Wealth index	Poor	3305(43.5)	723(29.9)	2582(49.9)	0.000	659(26.1)	2646(52.2)	0.000
	Middle	1588(20.9)	447(18.5)	1141(22.0)		448(17.8)	1140(22.5)	
	Rich	2697(35.5)	1245(51.6)	1452(28.1)		1416(56.1)	1280(25.3)	
Distance to health facility	Not big problem	4825(63.6)	1768(73.2)	3057(59.1)	0.000	1833(72.7)	2992(59.1)	0.000
	Big problem	2764(36.4)	647(26.8))	2118(40.9)		690(27.3)	2074(40.9)	
Insurance	Not insured	7273(95.8)	2272(94.1)	5001(96.6)	0.000	2362(93.6)	4910(96.9)	0.000
	*Insured*	*318(4.2)*	*143(5.9)*	*175(3.4)*		*161(6.4)*	*156(3.1)*	

### Bivariate analysis of socio-demographic and obstetric characteristics

The joint and marginal probabilities of ANC and DC together with the odds ratios and chi-square *p*-value was presented in Table 8. Once the association between the two outcomes (ANC and DC) has been determined, the frequency distribution of each predictor for the different combinations of ANC and DC was done.

**Table 8:** Joint and marginal probability of ANC and DC

EDHS Years	ANC	Place of Delivery		Marginal ANC	Odds Ratio	X^2^-*p* -value
		Health facility	Home			
2000	Yes	250 (0.031)	581(0.073)	831(0.104)	15.81	0.000
	No	189(0.024)	6946 (0.872)	7135(0.896)		
Marginal DC		439 (0.055)	7527(0.945)	7966 (1)		
2005	Yes	275 (0.038)	614 (0.084)	889 (0.122)	12.54	0.000
	No	221 (0.03)	6187(0.85)	6408 (0.878)		
Marginal DC		496(0.068)	6801(0.932)	7297(1)		
2011	Yes	533 (0.067)	1002(0.127)	1535 (0.194)	7.41	0.000
	No	427 (0.054)	5946(0.752)	6373 (0.806)		
Marginal DC		960(0.121)	6948 (0.878)	7908(1)		
2016	Yes	1409 (0.86)	1006 (0.133)	2415(0.318)	5.10	0.000
	No	1115 (0.147)	4061(0.535)	5176(0.682)		
Marginal DC		2524 (0.332)	5067(0.668)	7591(1)		

### EDHS 2000

The frequency distribution of each level of the predictor over DC conditioned on ANC in the 2000 Ethiopian demographic and health survey was presented in Table 9. The highest proportion of antenatal care and health facility delivery was observed among women aged 25–29 years and in each age group majority of the mothers had no ANC and deliver at home. DC with ANC was the highest among mothers who attend secondary education and DC with no ANC is more common among mothers who did not attend formal education. Moreover, among mothers who had both ANC and DC, 84.9% were from urban whereas 94.0% of women having neither ANC nor DC were from rural areas.

**Table 9:** Frequency distribution of socio-demographic and obstetric characteristics for the different combinations of ANC and DC, EDHS 2000

*Variable*	*Categories*	*ANC and Health facility (%)*	*ANC and Home (%)*	*No ANC and Health facility (%)*	*No ANC and Home (%)*
Age	15–19	16(6.4)	29(5.0)	29(15.4)	399(5.7)
	20–24	56(22.4)	122(21.0)	37(19.7)	1511(21.8)
	25–29	87(34.8)	177(30.5)	58(30.9)	1698(24.4)
	30–34	38(15.2)	118(20.3)	31(16.5)	1306(18.8)
	35–39	34(13.6)	84(14.5)	17(9.0)	1083(15.6)
	40–44	13(5.2)	35(6.0)	12(6.4)	645(9.3)
	45–49	6(2.4)	15(2.6)	4(2.1)	303(4.4)
Mother’s education	No education	48(19.1)	355(61.2)	103(54.5)	6032(86.8)
	Primary	63(25.1)	156(26.9)	36(19.0)	748(10.8)
	Secondary	124(49.4)	68(11.7)	49(25.9)	160(2.3)
	Higher	16(6.4)	1(0.2)	1(0.5)	6(0.1)
Mother’s Occupation	Not working	96(38.4)	209(36.0)	68(36.0)	2407(34.7)
	Working	154(61.6)	371(64.0)	121(64.0)	4536(65.3)
Husband education	No education	23(9.4)	265(45.8)	62(34.4)	4805(69.6)
	Primary	58(23.8)	164(28.4)	59(32.8)	1613(23.4)
	Secondary	120(49.2)	125(21.6)	49(27.2)	441(6.4)
	Higher	43(17.6)	24(4.2)	10(5.6)	40(0.6)
Husband occupation	Not working	3(1.2)	9(1.6)	1(0.6)	26(0.4)
	Working	240(98.8)	569(98.4)	179(99.4)	6869(99.6)
Household head	Male	188(75.2)	492(84.7)	141(74.2)	5933(85.4)
	Female	62(24.8)	89(15.3)	49(25.8)	1013(14.6)
Marital status	Unmarried	43(17.1)	55(9.5)	47(24.9)	639(9.2)
	Married	208(82.9)	526(90.5)	142(75.1)	6308(90.8)
Religion	Orthodox	175(70.0)	283(48.6)	110(58.2)	3485(50.2)
	Catholic	1(0.4)	5(0.9)	2(1.1)	50(0.7)
	Protestant	28(11.2)	59(10.1)	24(12.7)	1118(16.1)
	Muslim	45(18.0)	227(39.0)	50(26.5)	2012(29.0)
	Others	1(0.4)	8(1.4)	3(1.6)	281(4.0)
Region	Tigray	19(7.6)	61(10.5)	7(3.7)	448(6.4)
	Afar	2(0.8)	4(0.7)	2(1.1)	76(1.1)
	Amhara	33(13.2)	70(12.0)	48(25.3)	2071(29.8)
	Oromo	53(21.2)	271(46.6)	69(36.3)	2664(38.4)
	Somalia	2(0.8)	1(0.2)	4(2.1)	78(1.1)
	Benishangul	4(1.6)	5(0.9)	5(2.6)	68(1.0)
	SNNP	41(16.4)	138(23.8)	31(16.3)	1479(21.3)
	Gambela	3(1.2)	4(0.7)	2(1.1)	13(0.2)
	Harari	3(1.2)	1(0.2)	2(1.1)	10(0.1)
	Addis Ababa	84(33.6)	22(3.8)	16(8.4)	25(0.4)
	Dire Dwa	6(2.4)	4(0.7)	4(2.1)	14(0.2)
Residence	Urban	213(84.9)	183(31.6)	90(47.4)	420(6.0)
	Rural	38(15.1)	397(68.4)	100(52.6)	6527(94.0)
Family size	1–3	49(19.5)	69(11.9)	40(21.3)	954(13.7)
	4–6	115(45.8)	287(49.4)	90(47.9)	3547(51.1)
	7 and above	8734.7	225(38.7)	58(30.9)	2446(35.2)
Birth order	First	86(34.4)	111(19.1)	85(45.0)	1080(15.5)
	2–3	93(37.2)	173(29.8)	38(20.1)	2062(29.7)
	4–5	41(16.4)	166(28.6)	31(16.4)	1464(21.1)
	6 and above	30(12.0)	131(22.5)	35(18.5)	2340(33.7)
Birth interval	< = 24	28(17.5)	86(18.4)	18(17.3)	1157(19.7)
	25–36	41(25.6)	179(38.2)	49(47.1)	2183(37.3)
	> = 37	91(56.9)	203(43.4)	37(35.6)	2519(43.0)
Media exposure	No	36(14.4)	301(51.8)	78(41.3)	5380(77.5)
	Yes	214(85.6)	280(48.2)	111(58.7)	1559(22.5)
Has mobile/Tel	No	216(86.4)	575(99.1)	186(98.4)	6932(99.8)
	Yes	34(13.6)	5(0.9)	3(1.6)	14(0.2)
Wealth index	Poor	57(22.8)	123(21.3)	77(40.5)	3308(47.7)
	Middle	12(4.8)	49(8.5)	24(12.6)	913(13.2)
	*Rich*	*181(72.4)*	*406(70.2)*	*89(46.8)*	*2715(39.1)*

#### EDHS 2005

Table 10 revealed the joint frequency distribution of ANC and DC over the different predictor variables, in the 2005 Ethiopian demographic and health surveys. Concerning to age of mothers, ANC and DC were jointly more practiced by women aged 25–29 years old compared to other age groups. On the other hand, home delivery with no antenatal care visit was less common among women aged 15–19 years. Antenatal care followed by delivery care is the highest in Addis Ababa whereas home delivery without antenatal care is the highest in Oromia. Institutional delivery with antenatal care is more common among women in urban compared to rural women (79.2% Vs 20.8%). Moreover, mothers with a rich wealth index had the highest proportion of having both ANC and DC compared to mothers who have a poor and middle wealth index, whereas mothers who had a poor wealth index had the highest proportion of having neither antenatal nor delivery care.

**Table 10:** Frequency distribution of socio-demographic and obstetric characteristics for the different combinations of ANC and DC, EDHS 2005

*Variable*	*Categories*	*ANC and Health facility (%)*	*ANC and Home (%)*	*No ANC and Health facility (%)*	*No ANC and Home (%)*
Age	15–19	8(2.9)	40(6.5)	17(7.7)	373(6.0)
	20–24	75(27.4)	158(25.7)	61(27.7)	1178(19.0)
	25–29	84(30.7)	172(28.0)	80(36.6)	1623(26.2)
	30–34	56(20.4)	126(20.5)	22(10.0)	1221(19.7)
	35–39	30(10.9)	62(10.1)	16(7.3)	1027(16.6)
	40–44	18(6.6)	38(6.2)	16(7.3)	504(8.1)
	45–49	3(1.1)	18(2.9)	8(3.6)	261(4.2)
Mother’s education	No education	60(21.8)	393(64.0)	110(49.5)	5163(83.4)
	Primary	53(19.3)	150(24.4)	73(32.9)	927(15.0)
	Secondary	134(48.7)	67(10.9)	34(15.3)	93(1.5)
	Higher	28(10.2)	4(0.7)	5(2.3)	4(0.1)
Mother’s Occupation	Not working	153(55.8)	368(60.3)	141(63.5)	4371(70.7)
	Working	121(44.2)	242(39.7)	81(36.5)	1811(29.3)
Husband education	No education	56(88.9)	289(77.5)	97(75.8)	4083(81.9)
	Primary	2(3.2)	13(3.5)	6(4.7)	215(4.3)
	Secondary	2(3.2)	30(8.0)	11(8.6)	341(6.8)
	Higher	3(4.8)	41(11.0)	14(10.9)	344(6.9)
Husband occupation	Not working	4(1.5)	16(2.6)	1(0.5)	35(0.6)
	Working	265(98.5)	591(97.4)	217(99.5)	6115(99.4)
Household head	Male	204(74.2)	506(82.4)	179(81.0)	5520(89.2)
	Female	71(25.8)	108(17.6)	42(19.0)	666(10.8)
Marital status	Unmarried	5(7.7)	43(10.4)	17(13.3)	556(10.2)
	Married	60(92.3)	371(89.6)	111(86.7)	4884(89.8)
Religion	Orthodox	177(64.6)	294(47.9)	120(54.1)	2668(43.1)
	Catholic	1(0.4)	2(0.3)	1(0.5)	71(1.1)
	Protestant	40(14.6)	118(19.2)	53(23.9)	1193(19.3)
	Muslim	54(19.7)	194(31.6)	39(17.6)	2088(33.8)
	Others	2(0.7)	6(1.0)	9(4.1)	166(2.7)
Region	Tigray	20(7.3)	66(10.8)	17(7.7)	377(6.1)
	Afar	3(1.1)	3(0.5)	1(0.5)	62(1.0)
	Amhara	30(10.9)	105(17.1)	49(22.2)	1672(27.0)
	Oromo	63(23.0)	211(34.4)	83(37.6)	2357(38.1)
	Somalia	10(3.6)	3(0.5)	9(4.1)	266(4.3)
	Benishangul	2(0.7)	5(0.8)	11(5.0)	50(0.8)
	SNNP	42(15.3)	203(33.1)	33(14.9)	1352(21.9)
	Gambela	2(0.7)	4(0.7)	3(1.4)	14(0.2)
	Harari	3(1.1)	0(0)	2(0.9)	9(0.1)
	Addis Ababa	93(33.9)	11(1.8)	11(5.0)	13(0.2)
	Drie Dawa	6(2.2)	2(0.3)	2(0.9)	14(0.2)
Residence	Urban	217(79.2)	128(20.9)	75(33.9)	212(3.4)
	Rural	57(20.8)	485(79.1)	146(66.1)	5974(96.6)
Family size	1–3	62(22.5)	88(14.3)	61(27.6)	697(11.3)
	4–6	141(51.3)	329(53.6)	106(48.0)	3061(49.5)
	7 and above	72(26.2)	197(32.1)	54(24.4)	2429(39.3)
Birth order	First	112(40.9)	113(18.4)	85(38.5)	879(14.2)
	2–3	107(39.1)	233(38.0)	64(29.0)	1682(27.2)
	4–5	23(8.4)	124(20.2)	35(15.8)	1509(24.4)
	6 and above	32(11.7)	143(23.3)	37(16.7)	2115(34.2)
Birth interval	< = 24	28(17.4)	94(18.8)	27(19.7)	1115(21.0)
	25–36	22(13.7)	164(32.7)	49(35.8)	1766(33.3)
	> = 37	111(68.9)	243(48.5)	61(44.5)	2424(45.7)
Media exposure	No	28(10.2)	277(45.3)	95(43.0)	4157(67.5)
	Yes	246(89.8)	334(54.7)	126(57.0)	2004(32.5)
Has mobile/Tel	No	191(69.5)	589(96.1)	205(92.8)	6161(99.6)
	Yes	84(30.5)	24(3.9)	16(7.2)	23(0.4)
Wealth index	Poor	12(4.4)	145(23.6)	35(15.8)	2875(46.5)
	Middle	8(2.9)	127(20.7)	23(10.4)	1427(23.0)
	Rich	254(92.7)	342(55.7)	163(73.8)	1885(30.5)
Distance to Health facility	Not big problem	234(85.4)	436(71.1)	129(58.4)	3789(61.2)
	*Big problem*	*40(14.6)*	*177(28.9)*	*92(41.6)*	*2398(38.8)*

#### EDHS 2011

The joint distribution of ANC and DC in the EDHS 2011 is presented in Table 11. It can be seen that the minimum prevalence of joint ANC and DC was observed in the highest age groups (45–49) of the mother. Women who had no formal education have the lowest prevalence of joint antenatal and delivery care and the highest prevalence of having neither antenatal nor delivery care compared to primary or higher education levels. Among women who had both ANC and DC; 6.8, 4.1 and 89.1% of women had poor, middle, and rich wealth indexes respectively. In addition 31.1, 20.3, and 48.7% of women who had antenatal care with home delivery had poor, middle, and rich wealth indexes respectively (see Table 11).

**Table 11:** Frequency distribution of socio-demographic and obstetric characteristics for the different combinations of ANC and DC, EDHS 2011

*Variable*	*Categories*	*ANC and Health facility (%)*	*ANC and Home (%)*	*No ANC and Health facility (%)*	*No ANC and Home (%)*
Age	15–19	27(3.3)	42(4.6)	39(7.3)	308(5.6)
	20–24	200(24.4)	186(20.4)	160(30.0)	1050(19.1)
	25–29	301(36.7)	259(28.4)	164(30.8)	1568(28.5)
	30–34	170(20.7)	164(18.0)	76(14.3)	1097(19.9)
	35–39	99(12.1)	171(18.8)	57(10.7)	876(15.9)
	40–44	22(2.7)	68(7.5)	23(4.3)	437(7.9)
	45–49	1(0.1)	22(2.4)	14(2.6)	163(3.0)
Mother’s education	No education	162(19.8)	526(54.7)	264(49.5)	4232(77.0)
	Primary	340(41.5)	345(37.8)	201(37.7)	1209(22.0)
	Secondary	193(23.5)	28(3.1)	43(8.1)	48(0.9)
	Higher	125(15.2)	13(1.4)	25(4.7)	10(0.2)
Mother’s Occupation	Not working	395(48.7)	382(42.4)	274(51.9)	2984(54.8)
	Working	416(51.3)	519(57.6)	254(48.1)	2458(45.2)
Husband education	No education	86(10.8)	376(41.4)	164(31.2)	3306(60.6)
	Primary	304(38.1)	429(47.2)	221(42.1)	1836(33.6)
	Secondary	227(28.4)	60(6.6)	88(16.8)	219(4.0)
	Higher	181(22.7)	43(4.7)	52(9.9)	96(1.8)
Husband occupation	Not working	12(1.5)	13(1.4)	14(2.7)	109(2.0)
	Working	779(98.5)	891(98.6)	510(97.3)	5305(98.0)
Household head	Male	390(73.2)	847(84.5)	306(71.7)	5068(85.2)
	Female	143(26.8)	155(15.5)	121(28.3)	878(14.8)
Marital status	Unmarried	61(11.4)	77(7.7)	61(14.3)	524(8.8)
	Married	472(88.6)	925(92.3)	366(85.7)	5422(91.2)
Religion	Orthodox	335(62.7)	439(43.9)	245(57.4)	2307(38.8)
	Catholic	7(1.3)	8(0.8)	10(2.3)	56(0.9)
	Protestant	82(15.4)	210(21.0)	82(19.2)	1389(23.4)
	Muslim	108(20.2)	328(32.8)	88(20.6)	2038(34.3)
	Others	2(0.4)	16(1.6)	2(0.5)	150(2.5)
Region	Tigray	51(9.6)	113(11.3)	25(5.9)	341(5.7)
	Afar	3(0.6)	6(0.6)	3(0.7)	66(1.1)
	Amhara	88(16.5)	178(17.8)	131(30.8)	1594(26.8)
	Oromo	133(24.9)	446(44.5)	170(39.8)	2367(39.8)
	Somalia	6(1.1)	8(0.8)	12(2.8)	171(2.9)
	Benishangul	4(0.7)	11(1.1)	13(3.1)	64(1.1)
	SNNP	84(15.7)	209(20.9)	49(11.5)	1292(21.7)
	Gambela	5(0.9)	5(0.5)	4(0.9)	17(0.3)
	Harari	5(0.9)	1(0.1)	2(0.5)	10(0.2)
	Addis Ababa	147(27.5)	22(2.2)	12(2.8)	13(0.2)
	Dire Dwa	8(1.5)	3(0.3)	5(1.2)	11(0.2)
Residence	Urban	411(77.1)	151(15.1)	218(51.1)	408(6.9)
	Rural	122(22.9)	851(84.9)	209(48.9)	5538(93.1)
Family size	1–3	136(25.6)	157(15.7)	118(27.6)	667(11.2)
	4–6	299(56.2)	501(50.0)	214(50.1)	3042(51.2)
	7 and above	97(18.2)	343(34.3)	95(22.2)	2237(37.6)
Birth order	First	211(39.6)	179(17.9)	160(37.5)	849(14.3)
	2–3	216(40.5)	287(28.7)	149(34.9	1810(30.4)
	4–5	73(13.7)	272(27.2)	54(12.6)	1414(23.8)
	6 and above	33(6.2)	263(26.3)	64(15.0)	1873(31.5)
Birth interval	< = 24	29(9.1)	126(15.4)	65(24.3)	958(18.8)
	25–36	49(15.5)	266(32.4)	54(20.1)	1894(37.2)
	> = 37	239(75.4)	428(52.2)	149(55.6)	2241(44.0)
Media exposure	No	37(6.9)	240(24.0)	101(23.7)	2793(47.1)
	Yes	496(93.1)	761(76.0)	326(76.3)	3136(52.9)
Has mobile/telephone	No	430(80.7)	979(97.9)	396(92.7)	5922(99.6)
	Yes	103(19.3)	21(2.1)	31(7.3)	23(0.4)
Wealth index	Poor	36(6.8)	311(31.1)	76(17.8)	3012(50.7)
	Middle	22(4.1)	203(20.3)	43(10.1)	1359(22.9)
	Rich	475(89.1)	487(48.7)	308(72.1)	1574(26.5)
Distance to Health facility	Not big problem	464(87.5)	754(75.2)	332(78.3)	3853(64.8)
	Big problem	66(12.5)	248(24.8)	92(21.7)	2092(35.2)
Health Insurance	Not insured	506(94.9)	994(99.3)	419(98.6)	5929(99.7)
	*insured*	*27(5.1)*	*7(0.7)*	*6(1.4)*	*15(0.3)*

#### EDHS 2016

The prevalence of different measures of joint ANC and DC among women aged 15–49 years for the EDHS 2016 is summarized in Table 12. In this survey year, the minimum and maximum prevalence of joint ANC and DC were observed in the age of 45–49 and 25–29 years. Among mothers who have both ANC and DC, 53.4% were working and 46.6% were nonworking. In addition, joint care was more practiced by Orthodox followers and home delivery with no antenatal care was more common among Muslim followers. As well as delivering care with antenatal care increases as the wealth index of a mother increases (see Table 12).

**Table 12:** Frequency distribution of socio-demographic and obstetric characteristics for the different combinations of ANC and DC, EDHS 2016

*Variable*	*Categories*		*ANC and Health facility (%)*	*ANC and Home (%)*	*No ANC and Health facility (%)*	*No ANC and Home (%)*
Age		15–19	63(4.5)	41(4.1)	82(7.4)	153(3.8)
		20–24	305(21.7)	149(14.8)	295(26.5)	716(17.6)
		25–29	457(32.5)	334(33.2)	321(28.8)	1053(25.9)
		30–34	296(21.0)	240(23.8)	202(18.1)	922(22.7)
		35–39	207(14.7)	136(13.5)	153(13.7)	710(17.5)
		40–44	65(4.6)	79(7.8)	44(3.9)	358(8.8)
		45–49	15(1.1)	28(2.8)	17(1.5)	147(3.6)
Mother’s education		No education	520(36.9)	636(63.2)	517(46.4)	3118(76.8)
		Primary	493(35.0)	335(33.3)	434(38.9)	887(21)
		Secondary	232(16.5)	32(3.2)	114(10.2)	42(1.0)
		Higher	164(11.6)	3(0.3)	50(4.5)	13(0.3)
Mother’s Occupation		Not working	656(46.6)	550(54.7)	582(52.2)	2290(56.4)
		Working	753(53.4)	456(45.3)	533(47.8)	1770(43.6)
Husband education		No education	349(26.7)	439(47.0)	401(38.8)	2200(57.4)
		Primary	488(37.3)	420(45.0)	383(37.0)	1440(37.6)
		Secondary	254(19.4)	58(6.2)	155(15.0)	146(3.8)
		Higher	217(16.6)	17(1.8)	95(9.2)	46(1.2)
Husband occupation		Not working	73(5.6)	49(5.3)	81(7.8)	368(9.6)
		Working	1236(94.4)	884(94.7)	953(92.2)	3465(90.4)
Household head		Male	1137(80.7)	882(87.7)	947(84.9)	3509(86.4)
		Female	272(19.3)	124(12.3)	168(15.1)	552(13.6)
Marital status		Unmarried	100(7.1)	72(7.2)	81(7.3)	228(5.6)
		Married	1309(92.9)	933(92.8)	1034(92.7)	3833(94.4)
Religion		Orthodox	769(54.6)	355(35.3)	457(41.0)	1301(32.0)
		Catholic	5(0.4)	15(1.5)	8(0.7)	43(1.1)
		Protestant	262(18.6)	263(26.2)	239(21.5)	887(21.8)
		Muslim	369(26.2)	357(35.5)	397(35.6)	1701(41.9)
		Others	4(0.3)	15(1.5)	13(1.2)	128(3.2)
Region		Tigray	250(17.7)	54(5.4)	107(9.6)	126(3.1)
		Afar	7(0.5)	8(0.8)	7(0.6)	49(1.2)
		Amhara	274(19.4)	240(23.9)	243(21.8)	876(21.6)
		Oromo	321(22.8)	371(36.9)	471(42.3)	1966(48.4)
		Somali	16(1.1)	15(1.5)	37(3.3)	200(4.9)
		Benishangul	19(1.3)	15(1.5)	8(0.7)	39(1.0)
		SNNP	323(22.9)	288(28.7)	208(18.7)	782(19.3)
		Gambela	6(0.4)	3(0.3)	4(0.4)	8(0.2)
		Harari	5(0.4)	1(0.1)	5(0.4)	6(0.1)
		Addis Ababa	172(12.2)	5(0.5)	19(1.7)	2(0.0)
		Dire Dwa	17(1.2)	5(0.5)	5(0.4)	6(0.1)
Residence		Urban	560(39.7)	47(4.7)	256(23.0)	105(2.6)
		Rural	849(60.3)	958(95.3)	859(77.0)	3955(97.4)
Family size		1–3	291(20.7)	106(10.5)	227(20.4)	409(10.1)
		4–6	783(55.6)	529(52.6)	514(46.1)	2062(50.8)
		7 and above	335(23.8)	370(36.8)	374(33.5)	1590(39.2)
Birth order		First	458(32.5)	144(14.3)	357(32.0)	475(11.7)
		2–3	493(35.0)	315(31.3)	362(32.5)	1112(27.4)
		4–5	257(18.2)	268(26.6)	183(16.4)	1044(25.7)
		6 and above	202(14.3)	279(27.7)	212(19.0)	1430(35.2)
Birth interval		< = 24	128(13.5)	178(20.7)	125(16.6)	824(23.0)
		25–36	224(23.6)	243(28.3)	194(25.7)	1213(33.9)
		> = 37	597(62.9)	438(51.0)	436(57.7)	1546(43.1)
Media exposure		No	553(39.2)	688(68.5)	641(57.5)	3087(76.0)
		Yes	856(60.8)	317(31.5)	473(42.5)	974(24.0)
Has mobile/telephone		No	760(53.9)	865(86.1)	843(75.6)	3709(91.3)
		Yes	649(46.1)	140(13.9)	272(24.4)	352(8.7)
Wealth index		Poor	297(21.1)	426(42.3)	362(32.5)	2220(54.7)
		Middle	210(14.9)	237(23.6)	238(21.4)	903(22.2)
		Rich	901(64.0)	343(34.1)	514(46.1)	937(23.1)
Distance to Health facility		Not big problem	1108(78.6)	660(65.6)	725(65.0)	2332(57.4)
		Big problem	301(21.4)	346(34.4)	390(35.0)	1728(42.6)
Health Insurance		Not insured	1308(92.8)	964(95.8)	1054(94.6)	3946(97.2)
		*insured*	*101(7.2)*	*42(4.2)*	*60(5.4)*	*114(2.8)*

#### Regional antenatal and delivery care utilization

**Fig. 2:**
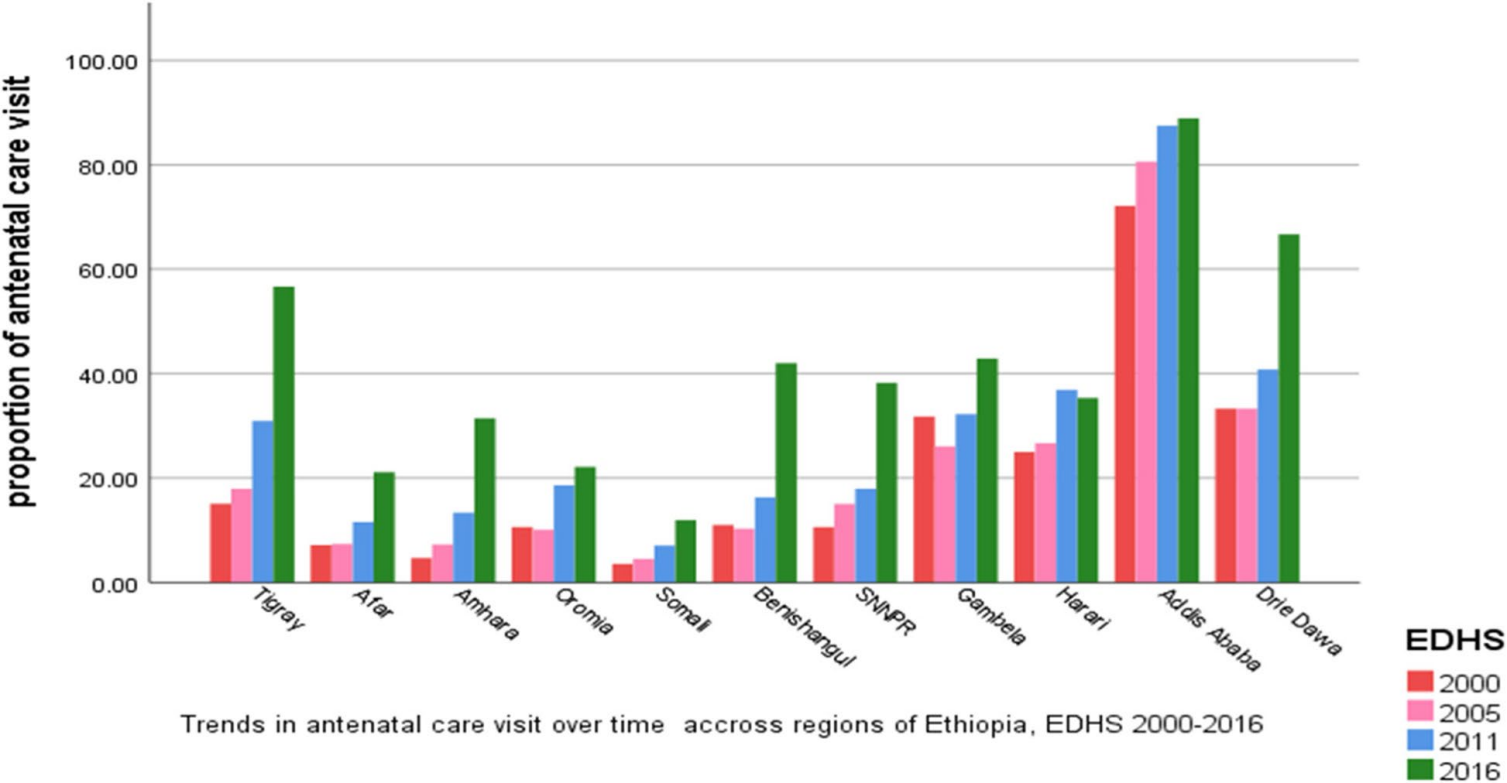
Regional Antenatal Care visit in Ethiopia EDHS 2000 to 2016

**Fig. 3:**
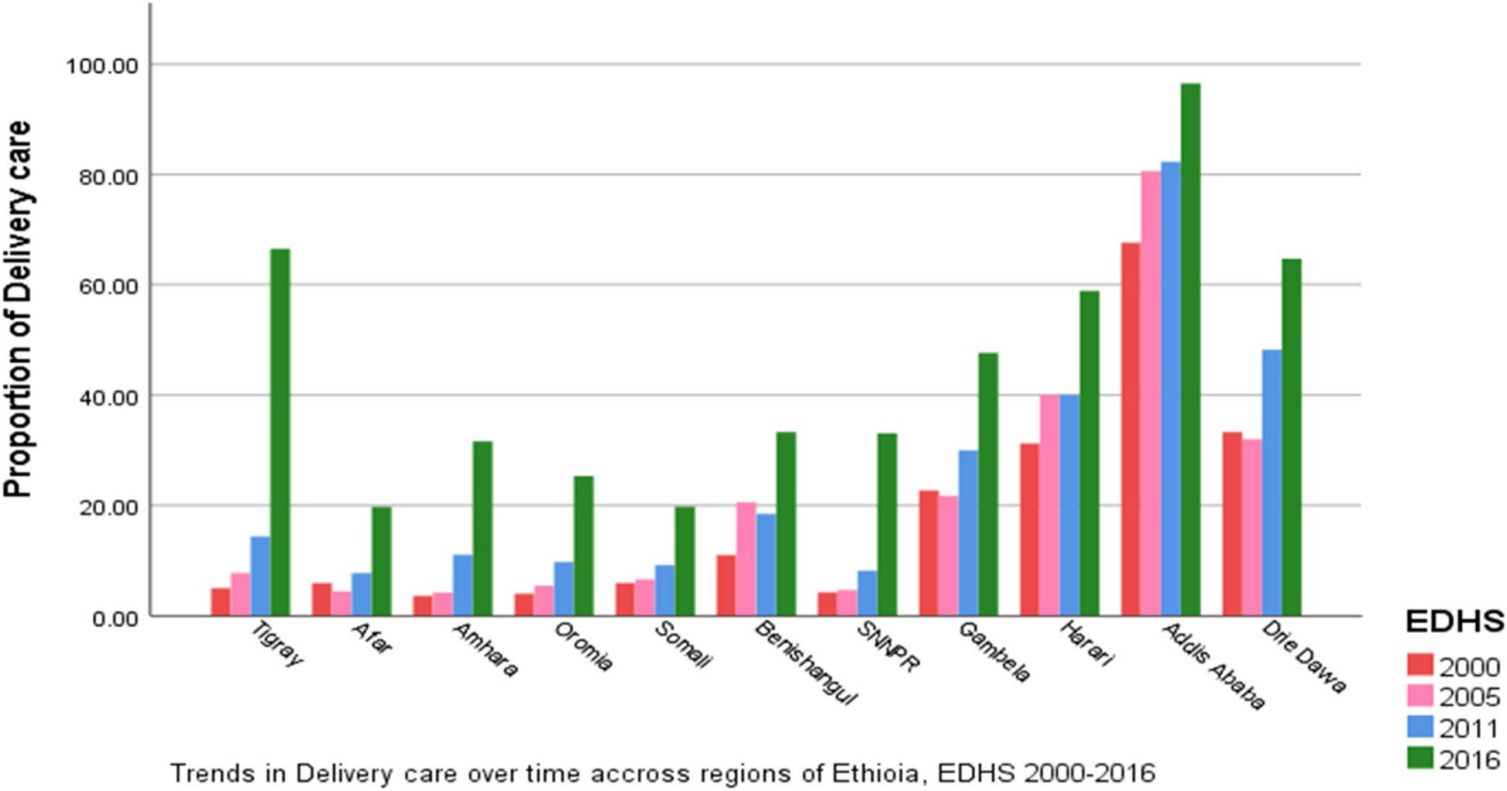
Regional Delivery Care in Ethiopia EDHS 2000 to 2016

As shown in Figs. 2 and 3 both ANC and DC varies across the region of the country. The highest ANC and DC were observed in Addis Ababa in all four survey years. Whereas the smallest proportion of ANC was observed in the Somali region in the four survey years. As well as a smaller proportion of DC was also seen in Amhara and Oromia regions. As shown by the figures, the two outcomes ANC and DC seem similar, thus we can suspect that they are associated over time across regions.

### Spatial analysis of antenatal and delivery care utilization

#### Spatial autocorrelation analysis of antenatal care

As presented in Table 13**,** the estimated Global Moran’s I in the four consecutive survey years (2000, 2005, 2011, and 2016) were 0.47369, 0.437928, 0.779599, and 0.402792 respectively with *p*-value < 0.00 l. This indicates that the spatial distribution of ANC was significantly clustered across EAs in all four survey years. Thus, it is likely hood that this clustered pattern could be a result of random chance.

**Table 13:** Indicator of spatial autocorrelation for ANC

EDHS	Statistic	z-score	*P*-value
2000	0.47369	61.455	< 0.001
2005	0.437928	55.834	< 0.001
2011	0.779599	73.568	< 0.001
2016	0.402792	41.017	< 0.001

#### Spatial distribution of antenatal care visit

**Fig. 4:**
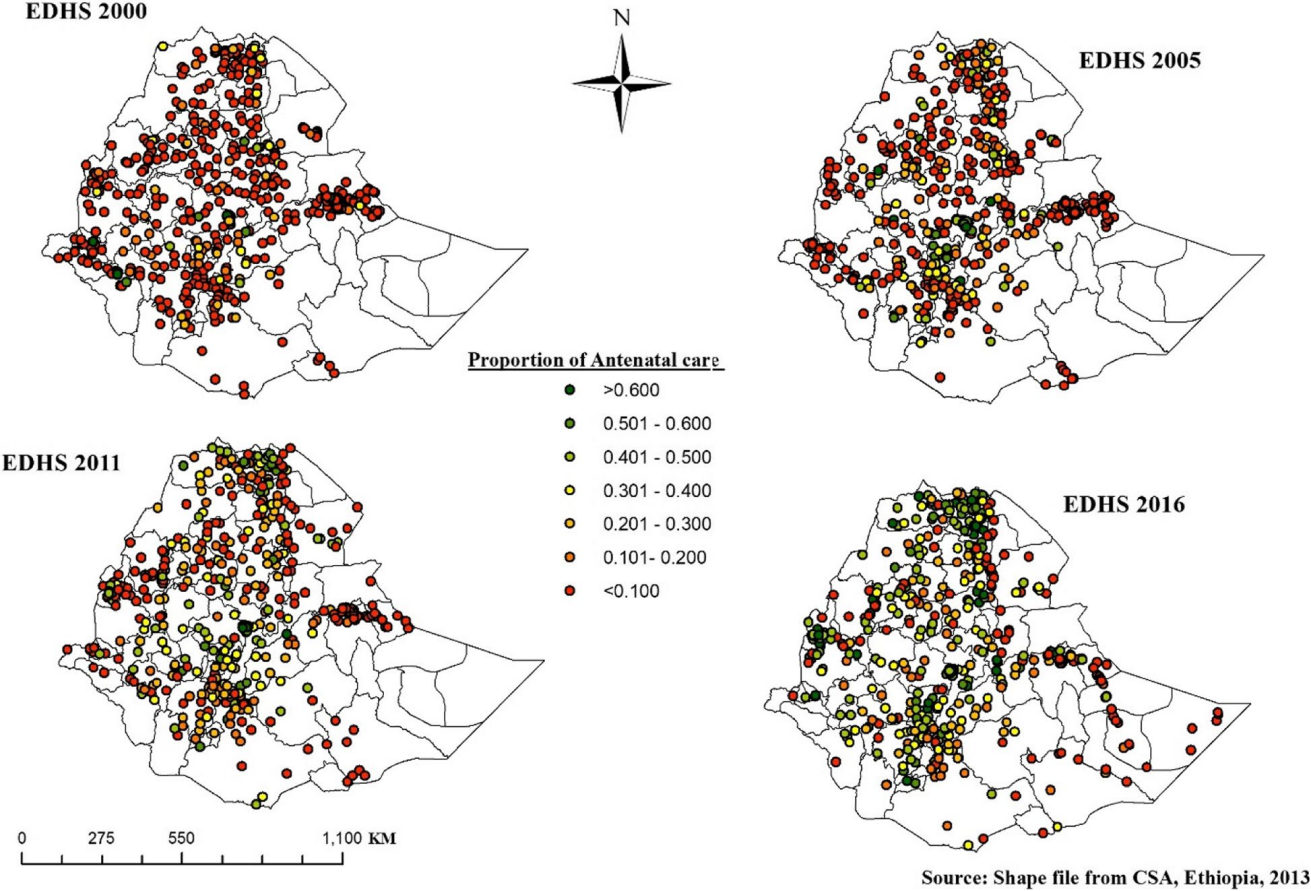
Spatial Distribution of ANC, EDHS 2000–2016

The proportion of ANC of each enumeration was represented by different colors and revealed in Fig. 4. Points with red color indicate enumeration areas with a low proportion of ANC and points with green color show an area that had a high proportion of ANC.

#### Hot spot analysis of antenatal care visit

**Fig. 5:**
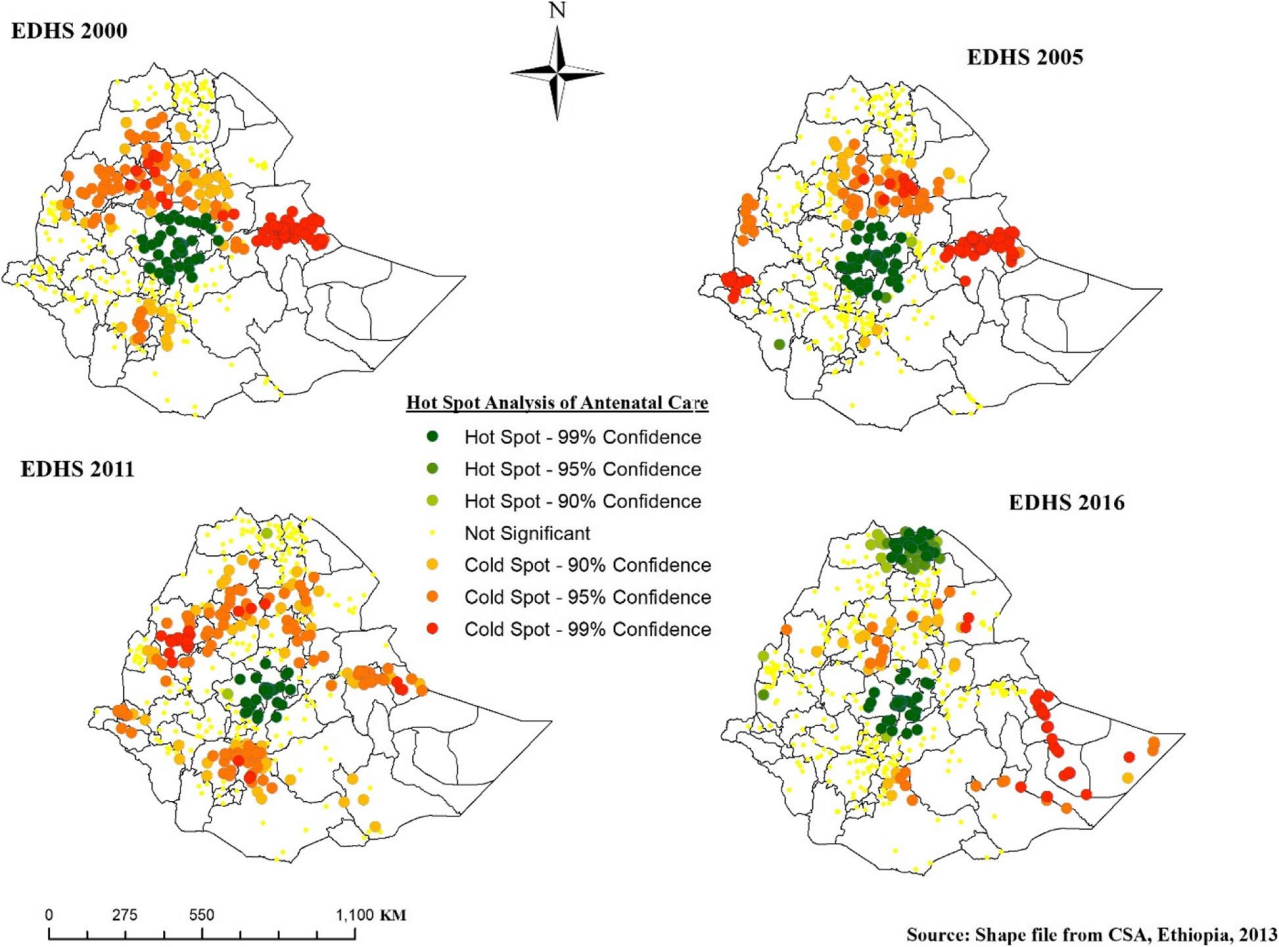
Hot Spot Analysis of ANC, EDHS 2000 to 2016

A point with green color in Fig. 5 indicates significant hot spot areas of ANC and was observed around Addis Ababa, North, and West Shewa, and west Hararge consistently in the four survey years and parts of central and western Tigray in the 2016 EDHS.

#### Spatial autocorrelation analysis of delivery care

Table 14 shows the Global Moran’s I report of DC in EDHS from 2000 to 2016. Given the values of the Moran I index, 0.420165 in 2000, 0.383160 in 2005, 0.741439 in 2011, and 0.395237in 2016 with *p*-values less than 0.05, indicates that geographically close EAs are more related than distant areas in the proportion of DC.

**Table 14:** Indicator of spatial autocorrelation for DC

EDHS	Statistic	z-score	*P*-value
2000	0.420165	61.455	< 0.001
2005	0.383160	55.83	< 0.001
2011	0.741439	73.568	< 0.001
2016	0.395237	41.017	< 0.001

#### Spatial distribution of delivery care

**Fig. 6:**
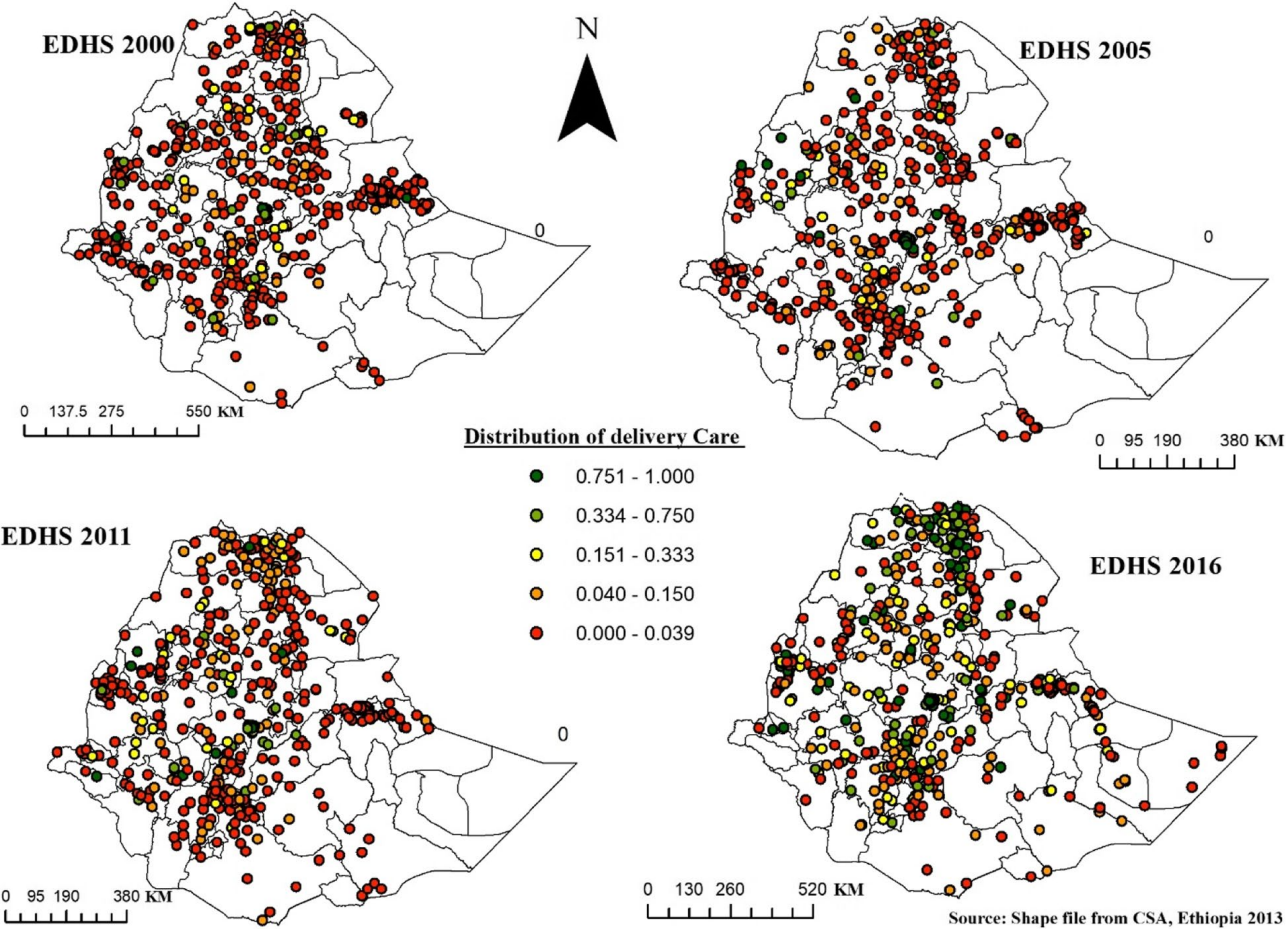
Spatial Distribution of DC in Ethiopia, EDHS 2000 to 2016

Figure 6 shows the spatial distribution of DC in Ethiopia from EDHS 2000 to 2016. The highest proportion of DC was represented by green color and was observed a little in central and eastern Tigray and Oromia special zone in 2000, somewhat around Metekel in 2005, highly in Northwestern, central, and Eastern Tigray in 2016, and Addis Ababa consistently in the four consecutive survey years. Whereas areas with a low proportion of DC were shaded as red and highly reported in most zones in the first three EDHS years.

#### Hot spot analysis of delivery care

**Fig. 7:**
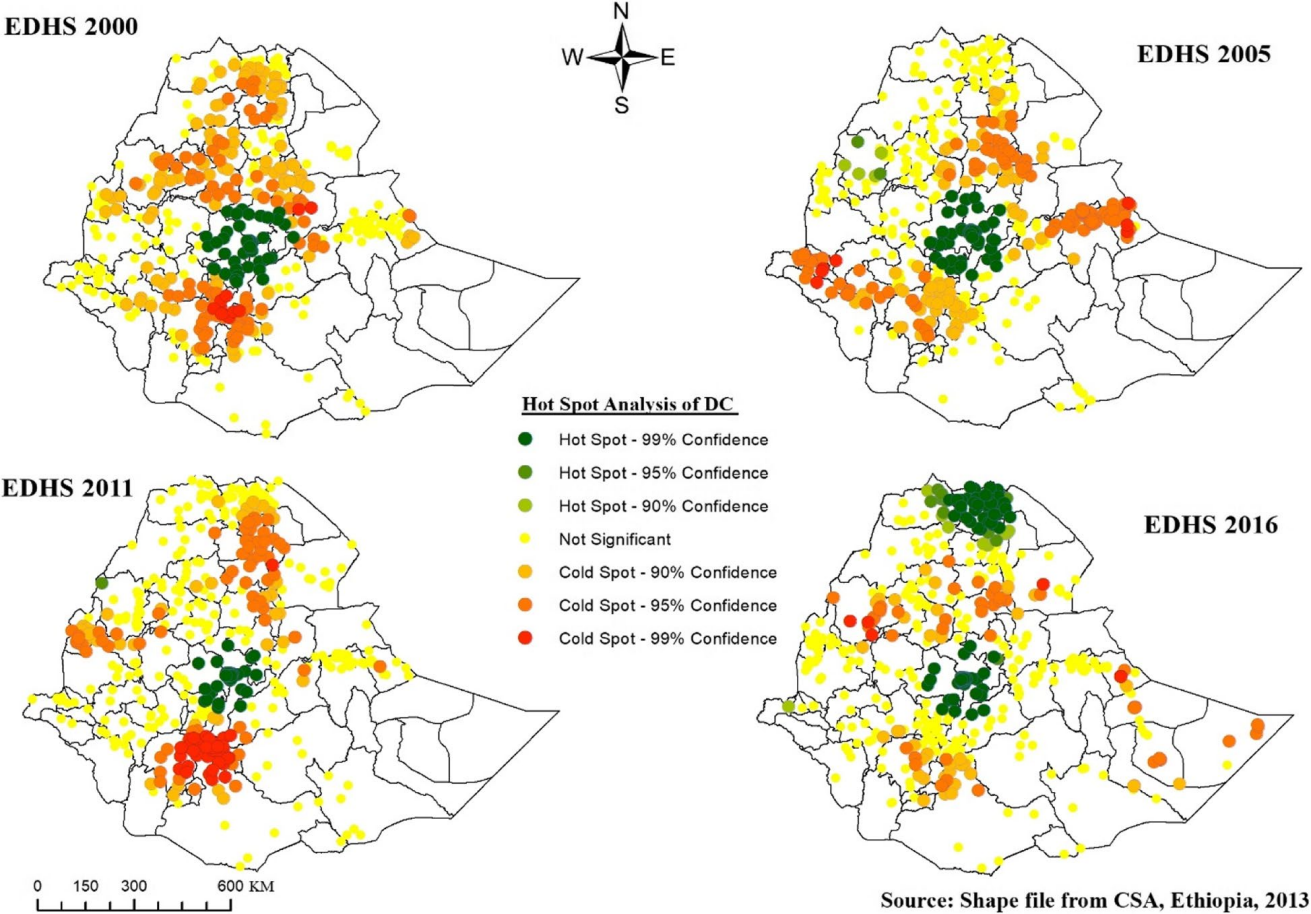
Hot Spot Analysis of DC in Ethiopia, EDHS 2000 to 2016

Areas with green points have high (hot spot) DC, which were observed around Addis Ababa in the first three survey years, and Addis Ababa and central and eastern Tigray in the last survey. Areas with red points are those that had a significant cold spot of DC, which were highly observed in North Gondar, East, and west Gojjam, North and South Wollo, Gurage, Dawaro, and Selt in 2000, East Hararge, Drie Dawa, Nur, Agnuak and South Wollo in 2005 and Gurage, Dawaro, Selt, West Arsi, Sidama in 2011 (see Fig. 7).

### Model fitting and parameters estimation

#### Bivariate binary logistic regression model for anc and dc

Table 15 presents the simultaneous effect of covariates on ANC and DC delivery. Taking into account the dependency of ANC and DC, a spatial bivariate binary logistic regression model was analyzed. The dependency between the two binary variables was measured by using the odds ratio (OR) equal to 2.029. Having checked the dependency of the two outcome variables ANC and DC, the effect of each predictor on ANC and DC was determined.

**Table 15:** Parameter estimates of the spatial bivariate binary logistic regression modeling of ANC and DC

*Variables*	Antenatal care visit (event = yes)		Place of Delivery (event = health facility)	
	Estimate (se.)	OR (95% CI)	Estimate(se)	OR(95% CI)
**Intercept**	-0.7482(0.2026)		1.0715(0.2133)	
**Age**				
15–19(ref)		1		1
20–24	0.07928(0.127)	1.0825 (0.843, 1.390)	-0.09237(0.136)	0.912 (0.700, 1.187)
25–29	0.2471(0.131)	1.280 (0.991, 1.654)	-0.06191(0.141)	0.940 (0.713, 1.239)
30–34	0.3743(0.138)	1.454 (1.109, 1.906)	0.1643(0.151)	1.179 (0.877, 1.584)
35–39	0.4419(0.144)	1.556 (1.172, 2.064)	0.1805(0.159)	1.198 (0.877, 1.636)
40–44	0.4048(0.158)	1.499 (1.099, 2.044)	0.1544(0.179)	1.167 (0.821, 1.660)
45–49	0.1888(0.189)	1.208 (0.834, 1.749)	0.2274(0.216)	1.255 (0.822, 1.916)
**Mother’s education**				
No education(ref)		1		1
Primary	0.4740(0.000)	1.606 (1.606, 1.606)	0.5015(0.059)	1.651 (1.470, 1.854)
Secondary	0.8835(0.094)	2.419 (2.011, 2.911)	1.0769(0.107)	2.935 (2.382, 3.618)
Higher	0.8552(0.163)	2.352 (1.708, 3.238)	1.5154(0.229)	4.551 (2.907, 7.125)
**Husband education**				
No education(ref)		1		1
Primary	0.3008(0.000)	1.351 (1.351, 1.351)	0.3238(0.059)	1.382 (1.230, 1.553)
Secondary	0.5814(0.074)	1.788 (1.546, 2.069)	0.9047(0.085)	2.471 (2.091, 2.920)
Higher	0.4415(0.098)	1.555 (1.283, 1.885)	0.7079(0.114)	2.0297 (1.624, 2.537)
**Husband occupation**				
Not working(ref)		1		1
Working	0.2987(0.099)	1.348 (1.110, 1.637)	0.2518(0.105)	1.286 (1.047, 1.580)
**Household head**				
Male	0.04214(0.056)	1.043 (0.934, 1.165)	-0.04013(0.066)	0.961 (0.844, 1.093)
Female (ref)		1		1
**Marital status**				
Unmarried (ref)		1		1
Married	0.00534(0.080)	1.0053 (0.860, 1.175)	-0.07839(0.093)	0.925 (0.771, 1.109)
**Religion**				
Orthodox (ref)		1		1
Catholic	-0.5361(0.216)	0.585 (0.383, 0.893)	-0.08831(0.269)	0.915(0.540, 1.551)
Protestant	-0.7095(0.074)	0.492 (0.426, 0.568)	-0.4699(0.092)	0.625 (0.522, 0.748)
Muslim	0.06931(0.059)	1.072 (0.955, 1.203)	0.03233(0.072)	1.033 (0.896, 1.190)
Others	-1.0357(0.183)	0.355 (0.248, 0.508)	0.1951(0.176)	1.215 (0.861, 1.715)
**Region**				
Tigray (ref)		1		1
Afar	-1.5083(0.117)	0.221 (0.176, 0.278)	-1.9130(0.149)	0.148 (0.110, 0.198)
Amhara	-0.9085(0.080)	0.403 (0.345, 0.472)	-0.8659(0.097)	0.421 (0.347, 0.509)
Oromo	-0.7804(0.082)	0.458 (0.390, 0.538)	-1.0351(0.101)	0.355 (0.291, 0.433)
Somalia	-1.7853(0.122)	0.168 (0.132, 0.213)	-1.3436(0.131)	0.261 (0.202, 0.337)
Benishangul	-0.2753(0.090)	0.759 (0.637, 0.905)	-0.05028(0.105)	0.951 (0.774, 1.168)
SNNP	-0.083(0.086)	0.920 (0.777, 1.089)	-0.6180(0.109)	0.539 (0.435, 0.668)
Gambela	0.01289(0.102)	1.013 (0.829, 1.237)	-0.4796(0.127)	0.619 (0.482, 0.794)
Harari	-0.9170(0.109)	0.400 (0.323, 0.495)	-0.1371(0.125)	0.872 (0.683, 1.113)
Addis Ababa	0.8101(0.127)	2.248 (1.752, 2.884)	0.5215(0.141)	1.685 (1.278, 2.220)
Dire Dawa	-0.1116(0.108)	0.894 (0.724, 1.104)	0.05091(0.129)	1.052 (0.817, 1.355)
**Residence**				
Urban (ref)		1		1
Rural	-0.9805(0.062)	0.375 (0.332, 0.424)	-1.8755(0.07043)	0.153 (0.134, 0.176)
**Birth order**				
First(ref)		1		1
2–3	0.1058(0.088)	1.112 (0.935, 1.322)	-0.2857(0.099)	0.751 (0.618, 0.913)
4–5	0.005187(0.102)	1.005 (0.824, 1.227)	-0.5720(0.116)	0.564 (0.450, 0.708)
6 and above	-0.2454(0.113)	0.782 (0.627, 0.976)	-0.6724(0.129)	0.510 (0.397, 0.657)
**Birth interval**				
< = 24 (ref)		1		1
25–36	0.05564(0.062)	1.057 (0.937, 1.193)	-0.1096(0.074)	0.896 (0.775, 1.036)
> = 37	0.2911(0.05863)	1.338 (1.193, 1.501)	0.2817(0.069)	1.325 (1.157, 1.519)
**Media exposure**				
No(ref)		1		1
Yes	0.5300(0.000)	1.699 (1.699, 1.699)	0.3731(0.055)	1.452 (1.303, 1.618)
**Has mobile/ Tele**				
No(ref)		1		1
Yes	0.1363(0.072)	1.146 (0.996, 1.319)	0.4556(0.077)	1.577 (1.357, 1.833)
**Wealth index**				
Poor(ref)		1		1
Middle	0.2968(0.059)	1.346 (1.197, 1.512)	0.1446(0.0724)	1.156 (1.003, 1.332)
Rich	0.5181(0.000)	1.679 (1.679, 1.679)	0.4158(0.062)	1.516 (1.343, 1.710)
**EDHS year**				
2000	-1.3455(0.073)	0.260 (0.226, 0.300)	-2.4807(0.084)	0.084 (0.071, 0.099)
2005	-1.3979(0.109)	0.247 (0.199, 0.306)	-2.2114(0.123)	0.110 (0.086, 0.139)
2011	-0.8638(0.062)	0.422 (0.373, 0.476)	-1.7262(0.072)	0.178 (0.154, 0.205)
2016 (ref)		1		1
*Si*	*0.4562(0.156)*	*1.578 (1.163, 2.142)*	*0.06245(0.153)*	*1.064 (0.788, 1.438)*

Measure of dependency Odds ratio = 2.029

Adjusting other predictors constant, the estimated odds of attending ANC among mothers aged 30–34 years was 1.454 times the estimated odds of attending ANC visits among mothers aged 15–19 years. Similarly, the estimated odds of attending ANC for mothers in age 40–44 years was 1.499 times the estimated odds of attending ANC among mothers aged 15–19 years. Ended place of delivery is not affected by age of the mothers.

The odds of women who attended secondary education attending ANC was 2.419 times the odds of none educated women attending ANC. Whereas the odds of those women who attended secondary education delivered at health facilities was 2.935 times that of the odds of none educated women delivered at health facilities. Likewise, mothers who had a primary educated partner were (1.351–1)100% = 35.1% and (1.382–1)100% = 38.2% time more likely to attend antenatal care visits and delivery at some health facility respectively, compared to mothers who had a none-educated partner (husband). Moreover, a mother with a working partner (husband) was (1.348–1)100% = 34.8 and (1.286–1)100% = 28.6% more likely to follow ANC and be delivered at health facilities respectively, given that other predictors remain constant.

The estimated odds of following ANC among mothers from middle and rich households were 1.346 and 1.679 times the estimated odds of following ANC among mothers from poor households respectively. This implies that middle and rich mothers were 34.6% and 67.9% more likely to attend antenatal care compared to poor mothers respectively. Likewise, the odds of institutional delivery among mothers from middle and rich households were 1.156 and 1.516 times the odds of institutional delivery among mothers from poor households respectively. From this, we can say that health facility delivery by middle and rich mothers was higher by 15.6% and 51.6% respectively compared to poor mothers. Generally, the odds of both ANC and DC increase as the household wealth index from which a mother came increases, given other variables remain constant.

The significant spatial variable with an appositive coefficient (0.4562) indicates that there was a spatial autocorrelation in the case of ANC between zones. This implies that zones with a high prevalence of ANC were usually surrounded by zones with a high prevalence of ANC and zones with a low prevalence of ANC were surrounded by zones with a low prevalence of ANC.

## Discussion 

This study revealed the association between antenatal and delivery care among mothers of reproductive age (15–49) in Ethiopia and determined the factors that jointly affect ANC and DC by considering spatial variability across zones in Ethiopia. The spatial bivariate binary logistic regression model was employed to evaluate the dependency between ANC and DC and to determine the associated factors.

This study revealed that the age of the mother has a significant effect on attending antenatal care visits. The odds of attending ANC among mothers aged 30–34, 35–39, and 40–44 years were 45.4%, 55.6%, and 49.95% more likely as compared to the odds that mothers aged 15–19 years attend antenatal care visits. This finding is consistent with studies conducted in East African Countries [12] and another study conducted in Ethiopia [12, 38]. Whereas the age of the mother has no significant association with the place of delivery. This finding is consistent with a study conducted in Ethiopia [22] but contradicts the study conducted in the Jhang district, Pakistan [39].

This study also revealed that the education level of mothers was significantly associated with antenatal care visits and it shows that the odds of attending ANC among mothers who attended primary, secondary and higher education were 1.606, 2.419, and 2.352 times the odds that women with no formal education attended ANC. This finding is consistent with the studies conducted in India [39], the Philippines and Indonesia, South East Asia [18] East African countries [12], and another study conducted in Ethiopia [38]. On the other hand, this finding contrasts the idea investigated in South West Shoa Zone, Ethiopia [40]. The findings of this study also show that the education level of mothers significantly affects the place where pregnant women delivered their chills. It revealed that women who attend primary, secondary, or higher education were more likely to deliver at health facilities and this is in line with the study conducted on determinants of institutional delivery service utilization in Ethiopia [22, 41, 42].

Husband education is another important variable that has a significant effect on the attendance of the recommended ANC among mothers in Ethiopia. The result shows that mothers who had a primary or more educated partner were more likely to deliver at a health facility as compared to mothers who had a non-educated partner. This finding supports the studies conducted in Nigeria [8] and another study conducted in Ethiopia [10, 38]. The finding of this study also revealed that partners’ education level also significantly affects the place of delivery of mothers. It shows that mothers who had a primary, secondary, and higher-educated partner (husband) were more likely to deliver at a health facility compared to mothers who had a non-educated partner. This result is in line with the result investigated in Ethiopia [22].

Based on the findings of this study husband education is one of the important variables that had a significant association with ANC. In this study, women whose husbands had some work were 34.8% more likely to have ANC as compared to mothers who had a non-working partner (husband). This result is consistent with the finding by [10]. Husband occupation also had a significant effect on the place of delivery of mothers in which, mothers who had a working partner were 28.6% more likely than mothers who had a non-working partner(husband) and this finding contrasts with the idea suggested by [22].

The finding of this study revealed that religion has a significant effect on ANC visits. The odds of ANC visits among Catholics, Protestants, and other followers were lower by 41.5%, 50.8%, and 64.5% respectively compared to Orthodox followers. This result is similar to the study investigated by [10]. Similarly, there is a significant difference in health facility delivery between Orthodox and protestant followers in that, protestant followers were 37.5% less likely to have health facility delivery compared to orthodox followers. This result is consistent with the result reported by [12, 22]. This might be due to the difference among mothers in their cultural and spiritual attitudes towards antenatal and delivery care service utilization.

Another crucial factor that significantly correlates with mothers' visits to the prenatal clinic in Ethiopia is where they live. Compared to mothers who originated from urban areas, women from rural regions were 62.5% less likely to visit. This is unquestionably due to the absence of medical facilities and competence in the region and their extensive experience. This finding supports the idea reported in India by [43], in the Philippines and Indonesia, in South East Asia by [18], in Nigeria [8], and in another study conducted in Ethiopia [10]. Whereas this finding contrasts with the finding in South West Shoa Zone, Ethiopia [40]. In addition place of residence also significantly affects place of delivery in which rural women were 84.7% less likely to deliver at a health facility compared to the odds that urban women deliver at a health facility. This finding is the same as with the studies conducted in the Jhang district, Pakistan [39], and Ethiopia [16]. The possible justification might be that women in an urban area easily get access to health knowledge and proximity to health facilities.

ANC visits among mothers who reside in middle and rich households were 34.6% and 67.9% respectively more likely than ANC among mothers who reside in poor households. This result supports the finding in Ethiopia [10, 38]. The result of this study also suggests that the household wealth index had a significant effect on the place of delivery among mothers in Ethiopia. The study shows that mothers who reside in middle and rich households were 15.6% and 51.6% more likely to deliver at a health facility compared to mothers who reside in poor households respectively. This result is consistent with the finding in the Jhang district, Pakistan [39], and Ethiopia [41]. This might be because financial problem leads to poor maternal health care.

This study incorporated data from four successive surveys and considered a simultaneous spatial variation of both antenatal and delivery care utilization. Thus, the findings generated from this research would improve the awareness of maternal healthcare utilization issues and will help policymakers implement appropriate policy measures. Apart from this, contribution, this study had several limitations. The survey in which the data were obtained for this study was obtained through five years intervals, i.e. 2000, 2005, 2011, and 2016. This leads to restricting the status of both delivery and antenatal care within five years. The DHS data are cross-sectional data and have a recall bias that can be mentioned as another limitation of the study. We recommend further studies using the latest survey data.

## Conclusion

Despite the government's claim that women now have better access to maternal health care, a sizable proportion of women continue to give birth at home without going to the advised antenatal care appointment. More than 87%, 85%, 75%, and 53% of women do not attend the recommended four or more ANC and deliver at home by the years 2000, 2005, 2011, and 2016 respectively. Low utilization of ANC and DC was observed around Fafan (Somali), East Hararge, South Gondar, West Gojjam, East Gojjam, South Wollo, South Gondar, Oromia special zone, Gamo Gofa, Sidama, and Gideo. Women and husbands with low education, having non-working partners, religion, regions of dwelling, residing in rural, lower birth order, low birth interval, unable to access mass media, low wealth status, and earlier EDHS survey years were significant predictors that hinder antenatal and delivery care utilization simultaneously in Ethiopia. Whereas the spatial variable significantly affects antenatal care and being unable to access mobile phones lead to low utilization of delivery care.

We recommend that policymakers, planners, and researchers consider these variables and the spatiotemporal distribution of ANC and DC to reduce maternal mortality in Ethiopia. Moreover, we recommend further studies using the latest EDHS survey data.

## Data Availability

The data that the authors used to produce this manuscript are available upon reasonable request from demographic and health survey (DHS) cite www.dhsprogram.com. The DHS Program is authorized to distribute, at no cost, unrestricted survey data files for legitimate academic research. Registration is required for access to data.
